# Burden and Future Trends of Gastric Cancer in 5 East Asian Countries From 1990 to 2036: Epidemiological Study Analysis Using the Global Burden of Diseases Study 2021

**DOI:** 10.2196/74389

**Published:** 2025-09-03

**Authors:** Tianhao Guo, Tingting Zhou, Wenjie Zhu, Yumo Yuan, Yifan Hui, Wenjian Zhu, Weixing Shen, Liu Li, Wei Wei, Haibo Cheng, Xiaoyu Wu

**Affiliations:** 1 Department of Surgical Oncology, Affiliated Hospital of Nanjing University of Chinese Medicine (Jiangsu Province Hospital of Chinese Medicine), 155 Hanzhong Road, Nanjing, Jiangsu, 210029, China, 86 13805168644; 2 Jiangsu Collaborative Innovation Center of Traditional Chinese Medicine Prevention and Treatment of Tumor, The First Clinical Medical College, Nanjing University of Chinese Medicine, Nanjing, China; 3 Institute of Health and Regimen, Jiangsu Open University, Nanjing, China; 4 Department of Gastroenterology, Wangjing Hospital of China Academy of Chinese Medical Sciences, Beijing, China; 5 School of Elderly Care Services and Management, Nanjing University of Chinese Medicine, Nanjing, China; 6 Department of Oncology, Affiliated Hospital of Nanjing University of Chinese Medicine, Nanjing, China

**Keywords:** gastric cancer, disease burden, global burden of disease study, epidemiological study, joinpoint regression analysis, age-period-cohort analysis, prediction model

## Abstract

**Background:**

Effective prevention and treatment are urgently needed, since gastric cancer (GC) poses a grave threat to the health and well-being of patients. The 5 East Asian countries (China, Japan, North Korea, South Korea, and Mongolia) represent one of the most significant regions globally in terms of GC burden.

**Objective:**

The goal of this study is to examine the patterns and trends of GC across 5 East Asian countries between 1990 and 2021.

**Methods:**

We retrieved data from the Global Burden of Disease Study (GBD) 2021 regarding the prevalence, incidence, mortality, years lived with disability (YLDs), years of life lost (YLLs), and disability-adjusted life years (DALYs) associated with GC in 5 East Asian countries from 1990 to 2021. We further assessed the burden of GC according to age and sex. We used decomposition analysis to examine the changes in the number of new cases, patients, and deaths related to GC. We also used Joinpoint (Joinpoint Regression Program, Version 5.1.0) and age-period-cohort analysis methods to interpret the epidemiological characteristics of GC. Autoregressive integrated moving average model (ARIMA) and Bayesian age-period-cohort (BAPC) prediction models were used to forecast the GC burden by 2036.

**Results:**

Among the 5 East Asian countries, China recorded the highest incidence, prevalence, death, YLLs, YLDs, and DALYs in both 1990 and 2021. From 1990 to 2021, the age-standardized rates for prevalence, mortality, incidence, YLDs, YLLs, and DALYs across the 5 East Asian countries showed an overall decline, though they remained higher than the global average. In all 5 East Asian countries, individuals aged 65 years and older consistently exhibited the highest rates for prevalence, incidence, mortality, YLDs, YLLs, and DALYs. The prevalence rate in South Korea, the incidence rate in North Korea and Mongolia, and the mortality rate in China are influenced by aging, surpassing the global aging average.

**Conclusions:**

The disease burden of GC in the 5 East Asian countries has consistently ranked high over the past 3 decades, particularly among the older individuals. The burden of GC in the 5 East Asian countries is expected to present a major public health challenge, primarily driven by the large population size and the aging demographic.

## Introduction

In 2022, gastric cancer (GC) accounted for 4.9% of all cancer cases, making it the fifth most commonly diagnosed cancer globally [1]. It is the fifth leading cause of cancer-related deaths, accounting for 6.8% of all cancer fatalities [1]. The incidence rate of GC remains high [2], particularly in Asian countries such as China [3,4]. It presents a significant challenge for both prevention and treatment, posing a serious threat to the life and health of affected individuals [5].

The 5 East Asian countries—China, Japan, North Korea, South Korea, and Mongolia—together account for about 22% of the global population and approximately 24% of the world’s gross domestic product (GDP) [6]. The 5 East Asian countries share similar geographical locations, cultural influences, and dietary and lifestyle habits. Their traditional medicines, including traditional Chinese medicine (TCM) and traditional Mongolian medicine, have complemented Western medicine effectively [7].

However, the existing GC registries remain insufficient, and epidemiological reports from the 5 East Asian countries are limited. The 2021 Global Burden of Diseases (GBD), Injuries, and Risk Factors Study performed a cause-of-death analysis, estimating mortality rates and years of life lost for 288 different causes of death [6]. This analysis categorized data based on age, sex, and location across 204 countries and regions, as well as 811 subnational areas, covering the period from 1990 to 2021 [6,8]. This study provides a comprehensive analysis of the disease burden of GC in 5 East Asian countries from 1990 to 2021, examining key metrics such as prevalence, incidence, mortality, years lived with disability (YLDs), years of life lost (YLLs), and disability-adjusted life years (DALYs). We also use the autoregressive integrated moving average (ARIMA) and Bayesian age-period-cohort (BAPC) models to predict the disease burden of GC to 2036. The aim of this study is to provide key insights that will assist decision-makers in assessing the overall burden of GC across the 5 East Asian countries. This, in turn, will support the formulation of targeted prevention strategies and help ensure the equitable distribution of public health resources.

## Methods

### Data Source

The Global Health Data Exchange GBD Results Tool was used to collect data spanning from 1990 to 2021. The GBD platform provides access to all age-standardized and age-specific rates, along with data that includes 95% uncertainty intervals. Potential issues with GBD data include variability in data quality across countries and years, which can affect comparability. Some regions have limited or incomplete health data, leading to reliance on statistical estimates and models that introduce uncertainty. In addition, differences in data collection methods and reporting standards may impact the accuracy and consistency of results. These factors should be considered when interpreting GBD findings.

We retrieved data from the GBD 2021 regarding the prevalence, incidence, mortality, YLDs, YLLs, and DALYs associated with GC of all ages in 5 East Asian countries (China, Japan, North Korea, South Korea, and Mongolia) from 1990 to 2021.

### Definition

In this study, we categorized all cancers coded as C16.902, following the *International Classification of Diseases, Tenth Revision* (*ICD-10*), as GC. By adhering strictly to *ICD-10* codes for cancer classification, the study ensured precise, reproducible identification of GC cases across different data sources and geographies, facilitating consistent burden measurement in GBD 2021.

### Data Analysis

The statistical methods used in GBD 2021 are described in detail in a separate source [6], including cause of death ensemble modeling, Bayesian Meta-regression, covariate inclusion, uncertainty quantification, and data standardization and adjustment, etc. Trends in prevalence, incidence, mortality, YLDs, YLLs, and DALYs from 1990 to 2021 were analyzed based on year, sex, and age group.

The average annual percent change (AAPC) and annual percentage change, along with their corresponding 95% CIs, were calculated using Joinpoint regression analysis (Joinpoint Regression Program, Version 5.1.0) to assess trends in the age-standardized prevalence rate (ASPR), age-standardized incidence rate (ASIR), age-standardized mortality rate (ASMR), age-standardized years lived with disability rate, age-standardized years of life lost rate, and age-standardized disability-adjusted life years rate (ASDR). All age-standardized rates (ASRs) were calculated using the age standard of the GBD world population.

The decomposition method uses several established approaches to analyze the differences in total deaths, attributing them to 3 main components: population growth, population aging, and changes in mortality rates [9-14].

The ARIMA model combines both the autoregressive and moving average models [15]. The ARIMA model is based on the assumption that the data series consists of time-dependent random variables, with their autocorrelation patterns described by the model. Consequently, future values can be predicted using past observations [16].

The BAPC model was used to predict the burden of GC up to 2036, using the BAPC and INLA packages in R. All analyses were performed using R (version 4.4.1, R Foundation) and Zstats v1.0 (Weijun Zheng), with a two-tailed *P* value of less than .05 deemed statistically significant [17].

### Ethical Considerations

The data used in this study are publicly accessible, and patient information was derived from aggregated data rather than individual-level records. This study aligns with an item considered exempt from review by the Ethics Committee of the Affiliated Hospital of Nanjing University of Chinese Medicine, specifically the category of “Previous data research.” Data were used in accordance with GBD’s terms of access for aggregated, anonymized data. As a result, the Ethics Committee of the Affiliated Hospital of Nanjing University of Chinese Medicine determined that the study was exempt from ethical review. All procedures performed in studies involving human participants were in accordance with the ethical standards of the institutional and national research committee and with the 1964 Helsinki declaration and its later amendments or comparable ethical standards. Informed consent was obtained from all individual participants included in the study.

## Results

### Overall Burden

In 1990, Japan had the highest ASPR among the 5 East Asian countries. In 2021, South Korea had the highest ASPR among the 5 East Asian countries. The ASPR of the 5 East Asian countries, except for North Korea, was above the global ASPR and the ASPR of Asia in 1990. The ASPR of North Korea was both below the global ASPR and the ASPR of Asia in 1990. The ASPR of the 5 East Asian countries was both above the global ASPR and the ASPR of Asia in 2021 (Table S1 in Multimedia Appendix 1).

In 1990, South Korea had the highest ASIR among the 5 East Asian countries. However, by 2021, Mongolia had the highest ASIR in the region. In 1990, the ASIR for all 5 East Asian countries, except North Korea, was higher than both the global and the Asian level. In contrast, the ASIR for North Korea was above the global ASIR but lower than the ASIR for Asia. By 2021, the ASIR for all 5 East Asian countries exceeded both the global ASIR and the ASIR for Asia (Table S2 in Multimedia Appendix 1).

In both 1990 and 2021, Mongolia had the highest ASMR among the 5 East Asian countries. The ASMR of the 5 East Asian countries, except for North Korea, was above the global ASMR and the ASMR of Asia in 1990. The ASMR of Japan and South Korea was above the global ASMR but below the ASMR of Asia in 2021 (Table S3 in Multimedia Appendix 1).

In 1990, Japan had the highest age-standardized YLDs rate among the 5 East Asian countries. In 2021, Mongolia had the highest age-standardized YLDs rate among the 5 East Asian countries. The age-standardized YLDs rate of the 5 East Asian countries except for North Korea was above the global and the Asian level in 1990. The age-standardized YLDs rate of North Korea was above the global but below the Asian level in 1990. The age-standardized YLDs rate of the 5 East Asian countries was both above the global and the Asian level in 2021 (Table S4 in Multimedia Appendix 1).

In both 1990 and 2021, Mongolia had the highest age-standardized YLLs rate and ASDR among the 5 East Asian countries. The age-standardized YLLs rate and ASDR of 5 East Asian countries were above the global and the Asian level in 1990. The age-standardized YLLs rate and ASDR of Japan and South Korea were above the global level but below the Asian level in 2021 (Table S5 and S6 in Multimedia Appendix 1).

### Descriptive Analysis From Age Perspective

In 2021, the age group with the highest prevalence numbers of females and males in China was 65‐69 years. The age group with the highest prevalence numbers of females in Japan was 80‐84 years, and the age group with the highest prevalence numbers of males in Japan was 70‐74 years. The age group with the highest prevalence numbers of females in South Korea was 80‐84 years, and the age group with the highest prevalence numbers of males in South Korea was 60‐64 years. The age group with the highest prevalence numbers of females in North Korea was 60‐64 years, and the age group with the highest prevalence numbers of males in North Korea was 55‐59 years. The age group with the highest prevalence numbers of females in Mongolia was 60‐64 years, and the age group with the highest prevalence numbers of males in Mongolia was 55‐59 years (Figure S1 in Multimedia Appendix 2).

In 2021, the age group with the highest incidence numbers of females in China was 70-74 years, and the age group with the highest incidence numbers of males in China was 65-69 years. The age group with the highest incidence numbers of females in Japan was 85-89 years, and the age group with the highest incidence numbers of males in Japan was 70-74 years. The age group with the highest incidence numbers of females in South Korea was 80-84 years, and the age group with the highest incidence numbers of males in South Korea was 75-79 years. The age group with the highest incidence numbers of females in North Korea was 75-79 years, and the age group with the highest incidence numbers of males in North Korea was 60-64 years. The age group with the highest incidence numbers of females and males in Mongolia was both 60-64 years (Figure S2 in Multimedia Appendix 2).

In 2021, the age group with the highest mortality numbers of females and males in China was both 70‐74 years. The age group with the highest mortality numbers of females in Japan was 85‐89 years, and the age group with the highest mortality numbers of males in Japan was 80‐84 years. In 2021, the age group with the highest mortality numbers of females and males in South Korea was both 80‐84 years. The age group with the highest mortality numbers of females in North Korea was 75‐79 years, and the age group with the highest mortality numbers of males in North Korea was 60‐64 years. The age group with the highest mortality numbers of females in Mongolia was 75‐79 years, and the age group with the highest mortality numbers of males in Mongolia was 60‐64 years (Figure S3 in Multimedia Appendix 2).

In 2021, the age group with the highest YLDs numbers of females in China was 70‐74 years, and the age group with the highest YLDs numbers of males in China was 65‐69 years. The age group with the highest YLDs numbers of females in Japan was 85‐89 years, and the age group with the highest YLDs numbers of males in Japan was 70‐74 years. The age group with the highest YLDs numbers of females in South Korea was 80‐84 years, and the age group with the highest YLDs numbers of males in South Korea was 60‐64 years. The age group with the highest YLDs numbers of females in North Korea was 60‐64 years, and the age group with the highest YLDs numbers of males in North Korea was 55‐59 years. The age group with the highest YLDs numbers of females and males in Mongolia was both 60‐64 years (Figure S4 in Multimedia Appendix 2).

In 2021, the age group with the highest YLLs and DALYs numbers of females and males in China was 65‐69 years. The age group with the highest YLLs and DALYs numbers of females in Japan was 85‐89 years, and the age group with the highest YLLs and DALYs numbers of males in Japan was 70‐74 years. The age group with the highest YLLs and DALYs numbers of females in South Korea was 80‐84 years, and the age group with the highest YLLs and DALYs numbers of males in South Korea was 60‐64 years. The age group with the highest YLLs and DALYs numbers of females in North Korea was 60‐64 years, and the age group with the highest YLLs and DALYs numbers of males in North Korea was 55‐59 years. The age group with the highest YLLs and DALYs numbers of females in Mongolia was 60‐64 years, and the age group with the highest YLLs and DALYs numbers of males in Mongolia was 55‐59 years (Figures S5 and S6 in Multimedia Appendix 2).

**Figure 1.**
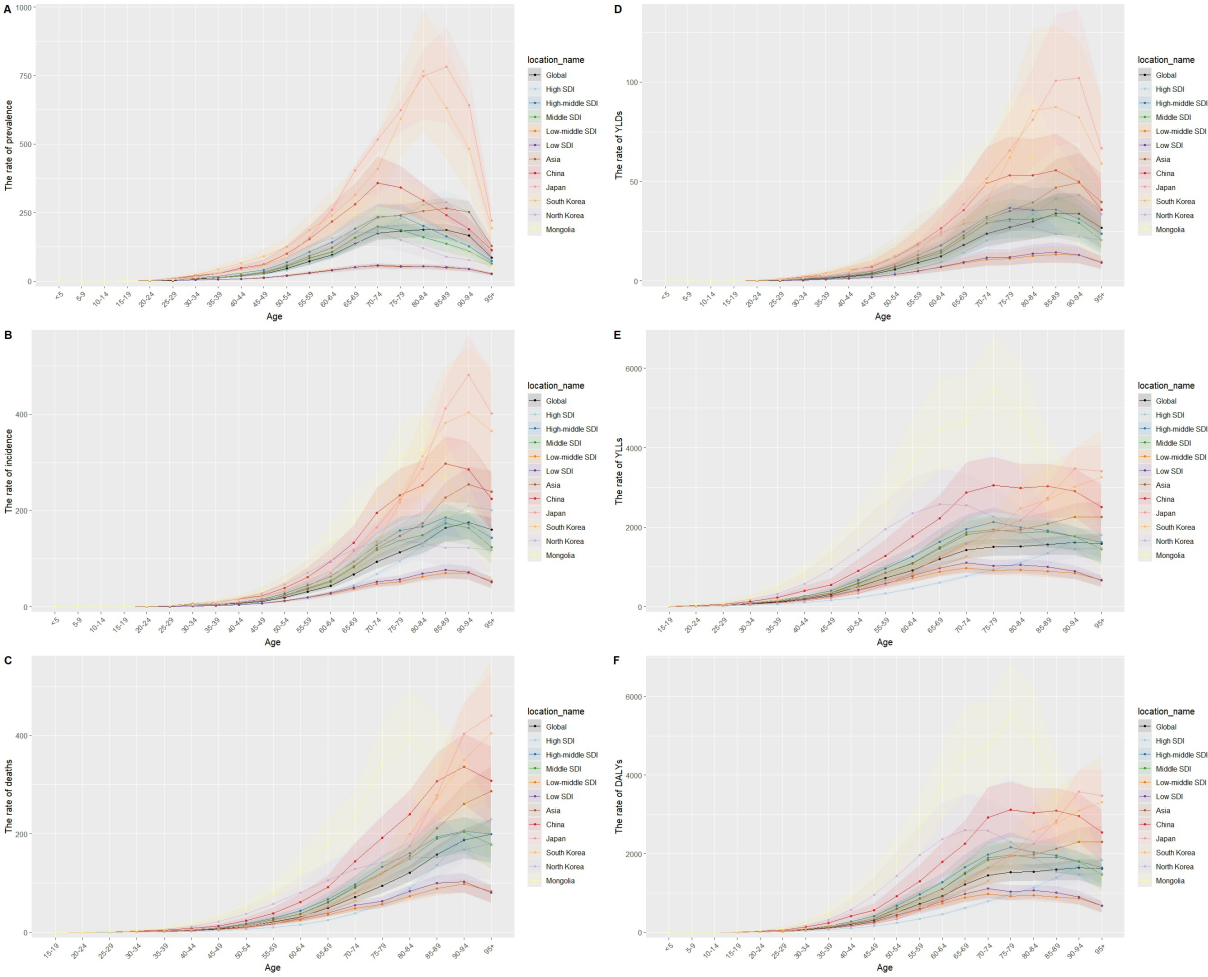
Age-specific rate in 5 East Asian countries. (A) The rate of prevalence. (B) The rate of incidence. (C) The rate of death. (D) The rate of YLDs. (E) The rate of YLLs. (F) The rate of DALYs. DALY: disability-adjusted life year; SDI: Sociodemographic Index; YLD: year lived with disability; YLL: year of life lost.

In 2021, the age group with the highest prevalence rate in China and North Korea was 70‐74 years. The age group with the highest prevalence rate in Japan was 85‐89 years. The age group with the highest prevalence rate in South Korea was 80‐84 years. The age group with the highest prevalence rate in Mongolia was 75‐79 years (Figure 1A).

In 2021, the age group with the highest incidence rate in China was 85‐89 years. The age group with the highest incidence rate in Japan and South Korea was 90‐94 years. The age group with the highest incidence rate in North Korea was 75‐79 years. The age group with the highest incidence rate in Mongolia was 80‐84 years (Figure 1B).

In 2021, the age group with the highest mortality rate in China was 90‐94 years. The age group with the highest mortality rate in Japan, South Korea, and North Korea was above 95 years. The age group with the highest mortality rate in Mongolia was 80‐84 years (Figure 1C).

In 2021, the age group with the highest YLDs rate in China and South Korea was 85‐89 years. The age group with the highest YLDs rate in Japan was 90‐94 years. The age group with the highest YLDs rate in North Korea was 70‐74 years. The age group with the highest YLDs rate in Mongolia was 75‐79 years (Figure 1D).

In 2021, the age group with the highest YLLs and DALYs rate in China and Mongolia was 75‐79 years. The age group with the highest YLLs and DALYs rate in Japan was 90‐94 years. The age group with the highest YLLs and DALYs rate in South Korea was above 95 years. The age group with the highest YLLs and DALYs rate in North Korea was 65‐69 years (Figure 1E and F).

### Descriptive Analysis From a Period Perspective

**Figure 2.**
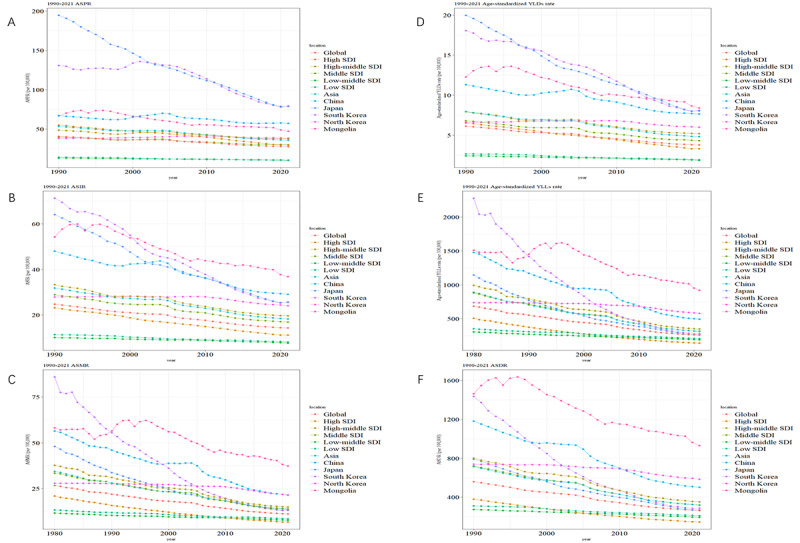
Time trends in 5 East Asian countries. (A) Age-standardized prevalence rate. (B) Age-standardized incidence rate (C) Age-standardized mortality rate. (D) Age-standardized YLDs rate. (E) Age-standardized YLLs rate. (F) Age-standardized DALYs rate. DALY: disability-adjusted life year; SDI: Sociodemographic Index; YLD: year lived with disability; YLL: year of life lost.

The ASPR, ASIR, ASMR, age-standardized YLDs rate, age-standardized YLLs rate, and ASDR of the 5 East Asian countries showed an overall downward trend from 1990 to 2021 (Figure 2). The ASPR, ASIR, ASMR, age-standardized YLDs rate, age-standardized YLLs rate, and ASDR for both females and males in the 5 East Asian countries showed an overall oscillating downward trend from 1990 to 2021 (Figures S7-12 in Multimedia Appendix 3).

The time trends of ASPR in Japan and South Korea ranked the top 2 among the 5 East Asian countries (Figure 2A). The time trends of ASIR in South Korea and Mongolia ranked the top 2 among the 5 East Asian countries (Figure 2B). The time trends of ASMR in South Korea and Mongolia ranked the top 2 among the 5 East Asian countries (Figure 2C). The time trends of YLDs in Japan, South Korea, and Mongolia ranked the top 3 among the 5 East Asian countries (Figure 2D). The age-standardized YLLs rate and ASDR in Mongolia ranked first among the 5 East Asian countries since 1990 (Figure 2E and F).

The ASPR in China and Mongolia was above the global level from 1990 to 2021 (Figure 2A). The ASPR in North Korea exceeded the global level in 1996 (Figure 2A). The ASIR, ASMR, age-standardized YLDs rate, age-standardized YLDs rate, age-standardized YLLs rate, and ASDR in the 5 East Asian countries were above the global level from 1990 to 2021 (Figure 2B-F).

### Joinpoint Regression Analysis

**Figure 3.**
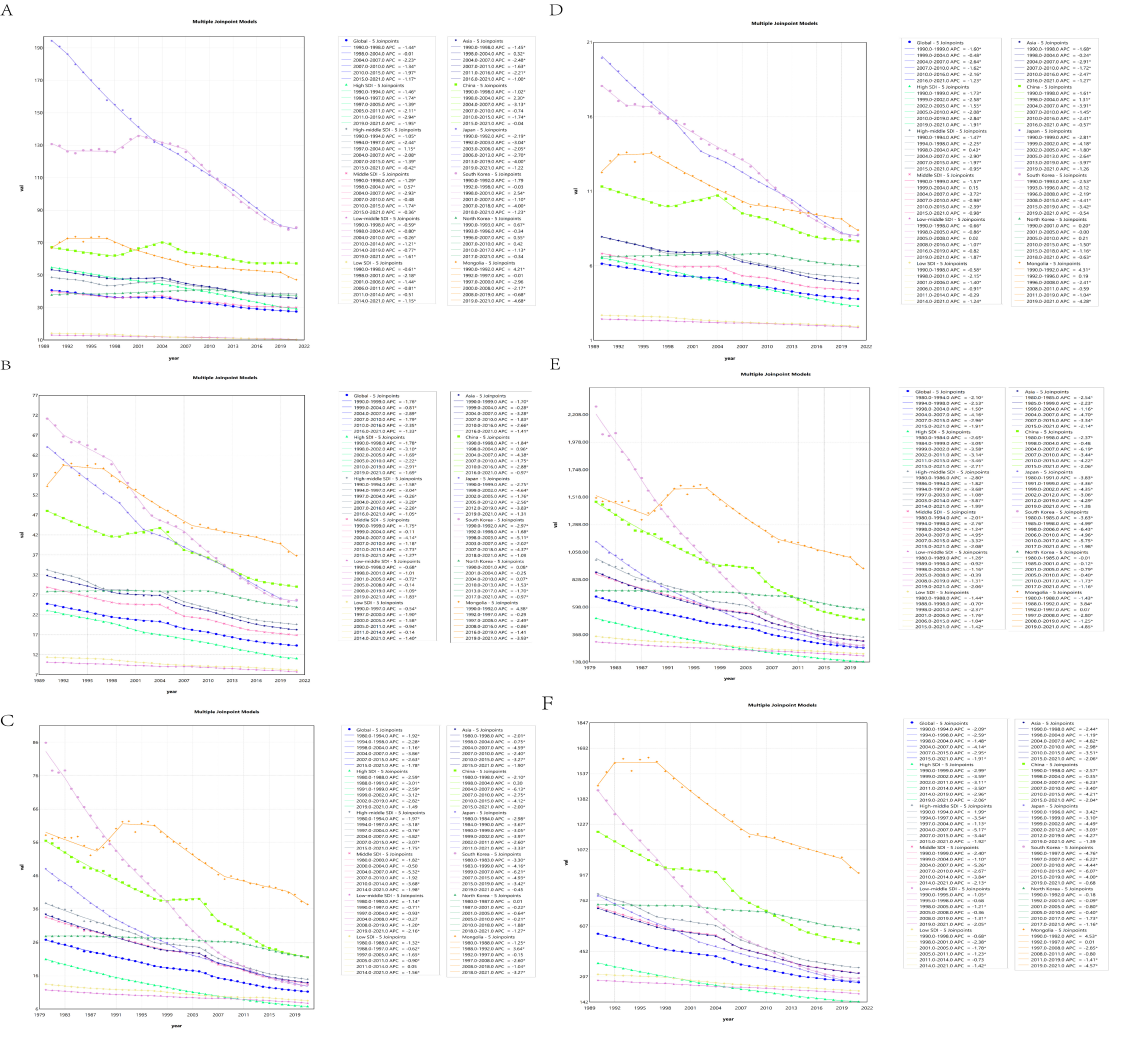
Joinpoint regression analysis of the age-standardized rate in 5 East Asian countries from 1990 to 2021. (A) Age-standardized prevalence rate. (B) Age-standardized incidence rate. (C) Age-standardized mortality rate. (D) Age-standardized YLDs rate. (E) Age-standardized YLLs rate. (F) Age-standardized DALYs rate. DALY: disability-adjusted life year; YLD: year lived with disability; YLL: year of life lost.

The AAPCs of ASPR in China (−0.50, 95% CI −0.67 to −0.32), North Korea (−0.05, 95% CI −0.16 to 0.05) and Mongolia (−1.14, 95% CI −1.44 to −0.84) were above the global level (−1.26, 95% CI −1.40 to −1.12). The AAPCs of ASPR in Japan (−2.92, 95% CI −3.11 to −2.72) and South Korea (−1.65, 95% CI −1.91 to −1.38) were below the global level (Figure 3A and Table S7 in Multimedia Appendix 4).

The AAPCs of ASIR in China (−1.61, 95% CI −1.73 to −1.48), North Korea (−0.48, 95% CI −0.52 to −0.44) and Mongolia (−1.26, 95% CI −1.60 to −0.92) were all above the global level (−1.76, 95% CI −1.89 to −1.64). The AAPCs of ASPR in Japan (−2.95, 95% CI −3.16 to −2.74) and South Korea (−3.23, 95% CI −3.47 to −2.98) were below the global level (Figure 3B and Table S7 in Multimedia Appendix 4).

The AAPCs of ASMR in North Korea (−0.62, 95% CI −0.65 to −0.60) and Mongolia (−1.11, 95% CI −1.47 to −0.75) were above the global level (−2.11, 95% CI −2.27 to −1.95). The AAPCs of ASMR in China (−2.34, 95% CI −2.60 to −2.07), Japan (−3.12, 95% CI −3.33 to −2.90) and South Korea (−4.47, 95% CI −4.65 to −4.28) were below the global level (see in Figure 3C and Table S7 in Multimedia Appendix 4).

The AAPCs of age-standardized YLDs rate in China (−1.25, 95% CI −1.38 to −1.12), North Korea (−0.33, 95% CI −0.37 to −0.28) and Mongolia (−1.25, 95% CI −1.57 to −0.93) were all above the global level (−1.57, 95% CI −1.70 to −1.44). The AAPCs of age-standardized YLDs rate in Japan (−2.93, 95% CI −3.14 to −2.71) and South Korea (−2.61, 95% CI −2.92 to −2.31) were below the global level (see Figure 3D and Table S7 in Multimedia Appendix 4).

The AAPCs of age-standardized YLLs rate in North Korea (−0.58, 95% CI −0.61 to −0.56) and Mongolia (−1.24, 95% CI −1.59 to −0.89) were above the global level (−2.36, 95% CI −2.55 to −2.17). The AAPCs of age-standardized YLLs rate in China (−2.65, 95% CI −2.86 to −2.43), Japan (−3.55, 95% CI −3.78 to −3.32) and South Korea (−4.95, 95% CI −5.17 to −4.73) were below the global level (see Figure 3E and Table S7 in Multimedia Appendix 4).

The AAPCs of ASDR in North Korea (−0.74, 95% CI −0.77 to −0.72) and Mongolia (−1.48, 95% CI −1.80 to −1.16) were above the global level (−2.42, 95% CI −2.52 to −2.33). The AAPCs of ASDR in China (−2.75, 95% CI −2.92 to −2.58), Japan (−3.43, 95% CI −3.61 to −3.24) and South Korea (−5.06, 95% CI −5.32 to −4.79) were below the global level (see Figure 3F and Table S7 in Multimedia Appendix 4).

### Age-Period-Cohort Analysis

The prevalence rate in China increased rapidly between the ages of 0 and 70 years, whereas the prevalence rate after age 70 years exhibited a gradual decline (Figure S13A in Multimedia Appendix 5). The prevalence rate in Japan increased rapidly between the ages of 0 and 80 years, while the prevalence rate after age 80 years displayed a gradual decline (Figure S13B in Multimedia Appendix 5). The prevalence rate in South Korea increased rapidly between the ages of 0 and 80 years, while the prevalence rate after age 80 years demonstrated a gradual decline (Figure S13C in Multimedia Appendix 5). The prevalence rate in North Korea rose rapidly between the ages of 0 and 70 years while the prevalence rate after 70 years showed a slowly decreasing trend (Figure S13D in Multimedia Appendix 5). In Mongolia, the prevalence rate surged significantly from ages 0 to 70 years, while the prevalence rate after age 70 years displayed a gradual decline (Figure S13E in Multimedia Appendix 5).

The incidence and death rate in China increased rapidly between the ages of 0 and 90 years, while the incidence and death rate after 90 years showed a slowly decreasing trend (Figure S14A-15A in Multimedia Appendix 5). In Japan, the incidence and death rate rose sharply from ages 0 to 90 years, while the incidence and death rate after age 90 years exhibited a gradual decline (Figure S14B-15B in Multimedia Appendix 5). The incidence and death rate in South Korea increased oscillably between the ages of 0 and 100 years (Figure S14C-15C Multimedia Appendix 5). In North Korea, the incidence and death rate surged significantly from ages 0 to 80 years, while the incidence and death rate after age 80 years exhibited a gradual decline (Figure S14D-15D in Multimedia Appendix 5). In Mongolia, the incidence and death rate rose sharply from ages 0 to 80 years, while the incidence and death rate after age 80 years displayed a gradual decline (Figure S14E-15E in Multimedia Appendix 5).

### Decomposition Analysis

**Figure 4.**
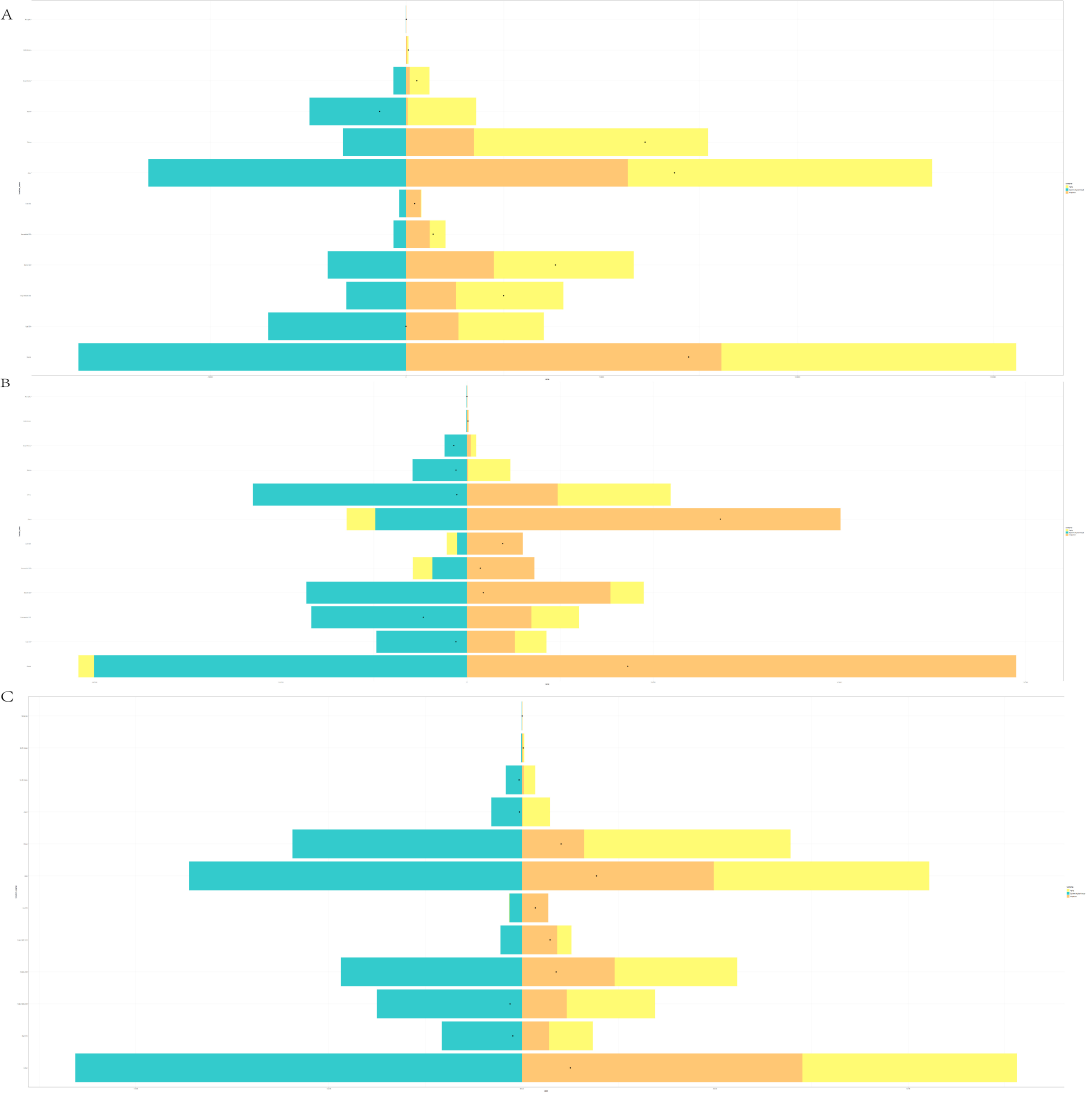
Decomposition analysis. (A) Prevalence. (B) Incidence. (C) Death.

The prevalence rate in China is 97.93% affected by aging, 28.41% affected by population, and −26.34% affected by epidemiological change. The prevalence rate in Japan is −258.68% affected by aging, −6.87% affected by population, and 365.55% affected by epidemiological change. The prevalence rate in South Korea is 183.07% affected by aging, 33.95% affected by population, and −117.02% affected by epidemiological change. The prevalence rate in North Korea is 61.85% affected by aging, 39.24% affected by population, and −1.09% affected by epidemiological change. The prevalence rate in Mongolia is 87.92% affected by aging, 94.71% affected by population, and −82.63% affected by epidemiological change (Figure 4A).

The incidence rate in China is −1108.04% affected by aging, −889.77% affected by population, and 2097.81% affected by epidemiological change. The incidence rate in Japan is −386.89% affected by aging, −10.57% affected by population, and 497.46% affected by epidemiological change. The incidence rate in South Korea is −41.33% affected by aging, −29.04% affected by population, and 170.37% affected by epidemiological change. The incidence rate in North Korea is 32.39% affected by aging, 135.40% affected by population, and −67.79% affected by epidemiological change. The incidence rate in Mongolia is 65.76% affected by aging, −1740.31% affected by population, and 1774.53% affected by epidemiological change (Figure 4B).

The death rate in China is 528.18% affected by aging, 159.04% affected by population, and −587.22% affected by epidemiological change. The death rate in Japan is −1075.28% affected by aging, −24.67% affected by population, and 1199.94% affected by epidemiological change. The death rate in South Korea is −382.19% affected by aging, −69.17% affected by population, and 551.36% affected by epidemiological change. The death rate in North Korea is 103.65% affected by aging, 78.25% affected by population, and −81.90% affected by epidemiological change. The death rate in Mongolia is 100.74% affected by aging, 154.25% affected by population, and −154.99% affected by epidemiological change (Figure 4C).

### ARIMA and BAPC Prediction

**Figure 5.**
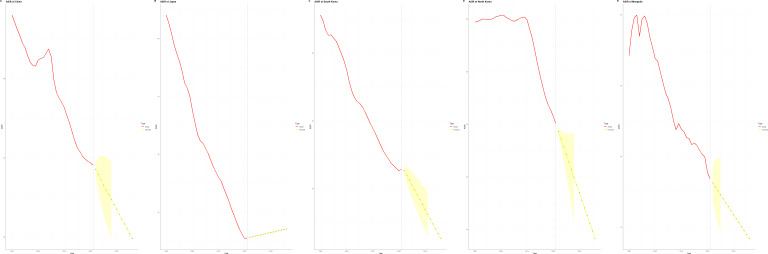
Autoregressive integrated moving average model prediction of age-standardized incidence rate. (A) China. (B) Japan. (C) South Korea. (D) North Korea. (E) Mongolia.

We conducted ARIMA and BAPC models to predict the situation up to 2036 (Figures5  6, S16-17 in Multimedia Appendix 6). The ASPR of China decreased from 1990 to 1997, increased from 1997 to 2004, and decreased after 2004. The ASPR of Japan demonstrated an overall downward trend from 1990 to 2036. The ASPR of South Korea gradually declined after reaching its peak around 2003 and rose again in 2021. The ASPR of North Korea gradually declined after reaching its peak around 2010 and rose again in 2024. The ASPR of Mongolia gradually declined after reaching its peak around 1993 and 1996 (Figure S16 and Table S8 in Multimedia Appendix 6).

**Figure 6.**
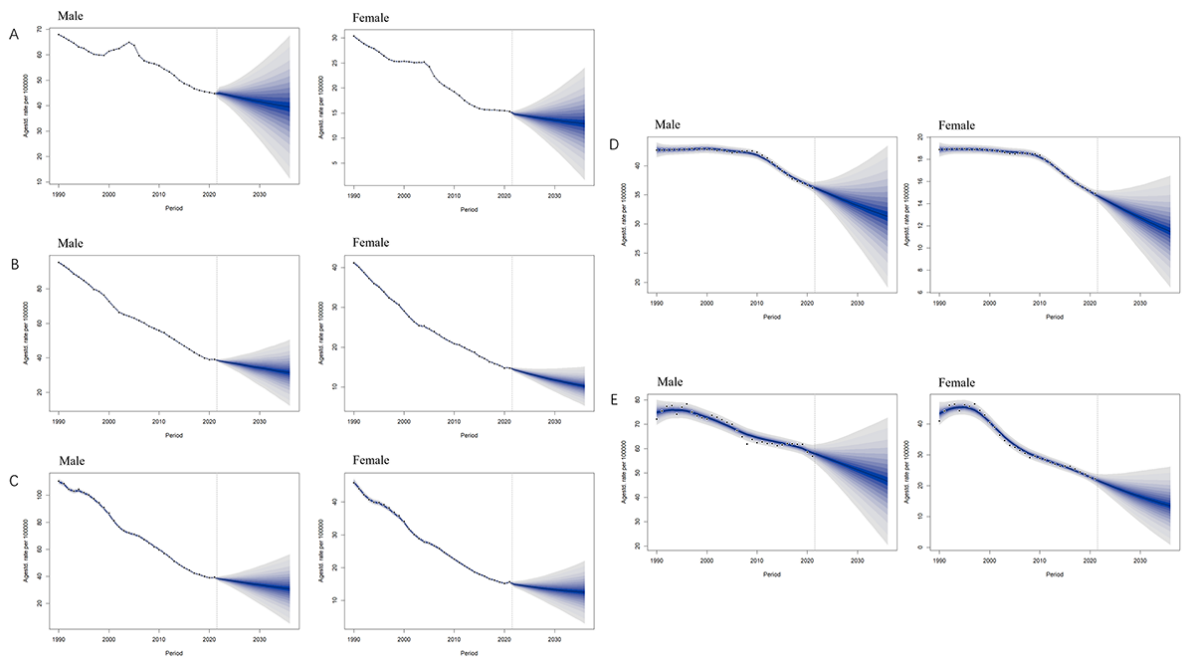
Bayesian age-period-cohort prediction of age-standardized incidence rate. (A) China. (B) Japan. (C) South Korea. (D) North Korea. (E) Mongolia.

The ASIR (Table 1) of China, South Korea, and North Korea demonstrated an overall downward trend from 1990 to 2036. The ASIR (Table 1) of Japan gradually declined and rose again in 2021. The ASIR (Table 1) of Mongolia gradually declined after reaching its peak around 1993 and 1996 (Figures5  6, and Table S9 in Multimedia Appendix 6).

**Table 1.** Autoregressive Integrated Moving Average prediction ASIR^a^ per 100,000 people in the 5 East Asian countries.

ASIR	China	Japan	South Korea	North Korea	Mongolia
1990	48.03	64.05	71.18	27.89	54.22
2021	29.05	25.54	25.76	24.02	36.83
2030	23.49	26.48	13.89	21.40	31.78
2036	19.80	27.11	5.30	19.65	28.41

a ASIR: age-standardized incidence rate.

The ASMR of China, Japan, South Korea, and North Korea demonstrated an overall downward trend from 1990 to 2036. The ASMR of Mongolia gradually declined after reaching its peak around 1993 and 1996 (Figure S17 and Table S10 in Multimedia Appendix 6).

## Discussion

### Principal Findings

This study offers a thorough analysis of the GC burden across 5 East Asian countries from 1990 to 2021. It highlights significant epidemiological trends and regional disparities, which are essential for guiding public health policies and optimizing resource allocation. Our findings indicate that China recorded the highest number of incidence, prevalence, death, YLLs, YLDs, and DALYs among the 5 East Asian countries in both 1990 and 2021. From an age perspective, in 2021, the age group with the highest prevalence numbers, incidence numbers, mortality numbers, YLD numbers, YLL numbers, and DALY numbers of females and males in the 5 East Asian countries was consistently those aged 55 and older. The age group with the highest prevalence rate, incidence rate, mortality rate, YLDs rate, YLLs rate, and DALYs rate in 5 East Asian countries was consistently those aged 65 years and older. The prevalence rate in South Korea was influenced by aging, surpassing global averages. The incidence rate in North Korea and Mongolia was influenced by aging and exceeds global averages. The mortality rate in China was influenced by aging, surpassing global averages. All of these factors indicate a significant disease burden on the older population.

In 1990, Japan had the highest ASPR among the 5 East Asian countries, but South Korea had the highest ASPR in 2021. In 1990, South Korea had the highest ASIR, and Japan had the highest age-standardized YLDs rate among the 5 East Asian countries, but Mongolia reported the highest ASIR and age-standardized YLDs rate in 2021. Mongolia also exhibited the highest ASMR, age-standardized YLLs rate, and ASDR among the 5 East Asian countries in both 1990 and 2021. The ASPR, ASIR, ASMR, age-standardized YLDs rate, age-standardized YLLs rate, and ASDR of the 5 East Asian countries demonstrated an overall downward trend from 1990 to 2021, but still higher than the global level. The AAPCs of ASPR, ASIR, ASMR, age-standardized YLDs rate, age-standardized YLLs rate, and ASDR in North Korea and Mongolia were above the global level. We used the ARIMA and BAPC models to project trends up to 2036. The ASIR and ASMR of Mongolia are predicted to be top 1 among 5 East Asian countries, while the ASPR of South Korea is predicted to be the highest. GC poses a substantial disease burden in 5 East Asian countries, particularly in China, Japan, South Korea, and Mongolia.

Gastrointestinal cancers account for nearly one-third of global cancer mortality [18]. GC demonstrated decreasing trends specifically in females from 2000 to 2019, but still at a high level [19]. The infection-noncommunicable disease pair with the largest burden was GC due to *Helicobacter pylori* [20]. The global incidence and mortality rates of both young- and late-onset GC have decreased since 1990, but the incidence rate of young-onset cancer has demonstrated a small but significant upward trend since 2015 [21]. From 1990 to 2019, the global incidence and mortality rates of GC declined in both sexes, across most GBD regions and countries, but the GC burden was still heavy in some GBD regions and countries, particularly within special age-specific groups [22]. In 2019, tracheal, bronchus, and lung (TBL), esophageal, stomach, colorectal, and pancreatic cancer were the top 5 neoplasms attributable to tobacco smoking, with different burdens in regions as per their development status [23]. The burden of stomach cancer worldwide, adjusted for age and related to smoking, has shown a decline from 1990 to 2019, but regional disparities have been identified, with some areas experiencing an increase in this burden [24]. ASIR and ASMR of GC in the total population, different regions, and countries in Asia from 1990 to 2019 showed an overall decreasing trend [25]. A study has found out that the GC burden attributed to high sodium intake still exists seriously and varies remarkably by regions, sex, and age across China [26]. Although the predicted age-standardized rates of mortality and DALYs due to dietary GC show downward trends, the absolute numbers are still predicted to increase in the next 25 years due to rapid population aging in China [27]. A considerable proportion of GC incidence might be preventable by healthy dietary habits in Korea [28].

GC susceptibility genes in East Asian populations differ from those in other regions [29-34]. To our knowledge, very few studies have focused on the epidemiological situation of GC in 5 East Asian countries. A study confirmed that ambient temperature has a significant impact on temperature-associated mortality, with cardiovascular mortality being especially vulnerable to both ambient temperature and diurnal temperature range [35].

Our study has several limitations. First, data within each iteration of GBD were comparable, but due to inherent limitations in the GBD methodology, results from different iterations, even for the same year, were not comparable. Differences in the GBD outcomes between iterations could be attributed to updates in data sources and improvements in methodology. Second, the accuracy of the estimated GC burden largely depends on the availability and quality of data acquired, which could be partially compensated by statistical methods. However, in regions with scarce data, especially in low-sociodemographic index countries, estimates could only rely on predictive covariates or global trends with consideration of sociodemographic index level and data from a single country, which is less representative. Extra caution should be taken when interpreting data from these areas. Additional high-quality population-based studies should be performed, especially in countries with scarce data. Third, some sources of uncertainty, including the covariates used in models, are not captured in our estimation process. The long-term impact of the COVID-19 pandemic may lead to deviation in the prediction model.

### Conclusions

The disease burden of GC in 5 East Asian countries has been consistently substantial over the past 3 decades, particularly among older people. Considering the complexity of GC pathogenesis, the focus of current research should be on the etiological research and the development of therapeutic strategies. Public health strategies need to enhance early diagnosis and prevention of GC, as well as optimize the regional distribution of health care resources and implement effective environmental health policies. Research on the epidemiology of GC in the 5 East Asian countries population should be strengthened, particularly focusing on differentiated management and precise prevention and control strategies for various regions, genders, and age groups. Meanwhile, rehabilitation and long-term care for patients with GC require greater attention to improve patients’ quality of life and reduce the burden on society and the health care system [36]. Complementary and alternative medicine combined with modern medicine plays an active role in the treatment of GC [37-39].

## Supplementary material

10.2196/74389 Multimedia Appendix 1 Prevalence, incidence, deaths, years lived with disability, years of life lost, and disability-adjusted life years of gastric cancer between 1990 and 2021 at the global and regional level and for 5 East Asian countries.

10.2196/74389 Multimedia Appendix 2 Age-specific prevalence, incidence, death, years lived with disability, years of life lost, and disability-adjusted life years numbers in 5 East Asian countries.

10.2196/74389 Multimedia Appendix 3 Trends in the all-age prevalence, incidence, death, years lived with disability, years of life lost, and disability-adjusted life years number and rate by sex from 1990 to 2021.

10.2196/74389 Multimedia Appendix 4 Average annual percent change of gastric cancer from 1990 to 2021 at the global and regional level and for 5 East Asian countries.

10.2196/74389 Multimedia Appendix 5 Age-period-cohort analysis of prevalence, incidence, and death rate.

10.2196/74389 Multimedia Appendix 6 Autoregressive integrated moving average model and Bayesian age-period-cohort prediction in the 5 East Asian countries.

## Abbreviations

AAPC: average annual percent change
ARIMA: autoregressive integrated moving average model
ASDR: age-standardized disability-adjusted life years rate
ASIR: age-standardized incidence rate
ASMR: age-standardized mortality rate
ASPR: age-standardized prevalence rate
BAPC: Bayesian age-period-cohort
DALY: disability-adjusted life year
GBD: Global Burden of Disease
GC: gastric cancer
*ICD-10*: *International Classification of Diseases, Tenth Revision*
YLD: year lived with disability
YLL: year of life lost
