# Supplementary material for: Burden and Future Trends of Gastric Cancer in 5 East Asian Countries From 1990 to 2036: Epidemiological Study Analysis Using the Global Burden of Diseases Study 2021
Source: JMIR Cancer. 2025 Sep 3;11:e74389. doi: 10.2196/74389 (PMC12408060; doi:10.2196/74389)
Supplement: Multimedia Appendix 1 [file cancer-v11-e74389-s001.docx]

Multimedia Appendix 1: Prevalence, incidence, deaths, YLDs (Years Lived with Disability), YLLs (Years of Life Lost), and DALYs (Disability-Adjusted Life Years) of gastric cancer between 1990 and 2021 at the global, regional and 5 East-Asian countries level.

Table of content

Table S1. Prevalence of gastric cancer between 1990 and 2021 at the global, regional and 5 East-Asian countries level.

Table S2. Incidence of gastric cancer between 1990 and 2021 at the global, regional and 5 East-Asian countries level.

Table S3. Deaths of gastric cancer between 1990 and 2021 at the global, regional and 5 East-Asian countries level.

Table S4. YLDs (Years Lived with Disability) of gastric cancer between 1990 and 2021 at the global, regional and 5 East-Asian countries level.

Table S5. YLLs (Years of Life Lost) of gastric cancer between 1990 and 2021 at the global, regional and 5 East-Asian countries level.

Table S6. DALYs (Disability-Adjusted Life Years) of gastric cancer between 1990 and 2021 at the global, regional and 5 East-Asian countries level.

**Table S1.** **Prevalence of gastric cancer between 1990 and 2021 at the global, regional and 5 East-Asian countries level.**

| Location | 1990 Prevalence cases (95% UI) |  |  | 1990 Age-standardized rates per 100 000 people (95% UI) |  |  | 2021 Prevalence cases (95% UI) |  |  | 2021 Age-standardized rates per 100 000 people (95% UI) |  |  |
| --- | --- | --- | --- | --- | --- | --- | --- | --- | --- | --- | --- | --- |
|  | Total | Male | Female | Total | Male | Female | Total | Male | Female | Total | Male | Female |
| Global | 1671262 (1524321,1796161) | 1097840 (955641,1216289) | 573423 (529307,619962) | 40.64 (37.25,43.68) | 56.74 (49.98,62.73) | 26.39 (24.29,28.53) | 2393213 (2059698,2771476) | 1698257 (1404777,2052649) | 694957 (606527,783245) | 27.58 (23.75,31.89) | 41.41 (34.19,49.83) | 15.23 (13.29,17.16) |
| SDI |  | | | | | | | | | | | |
| High SDI | 592944 (569778,608932) | 384563 (370025,395544) | 208380 (193742,216583) | 54.75 (52.72,56.23) | 80.21 (77.01,82.47) | 34.37 (32.14,35.61) | 592767 (542982,629774) | 403583 (375773,434926) | 189184 (161485,206665) | 29.39 (27.2,31.24) | 43.07 (40.18,46.57) | 17.51 (15.48,18.83) |
| High-middle SDI | 500512 (445395,544263) | 330709 (275907,372144) | 169804 (154923,186623) | 48.66 (43.46,52.91) | 71.35 (59.94,80.2) | 30.12 (27.42,33.11) | 749747 (610195,903200) | 549250 (418350,689455) | 200497 (170512,236752) | 38.28 (31.13,46.09) | 60.09 (45.91,75.05) | 19.22 (16.32,22.78) |
| Middle SDI | 452701 (385138,524307) | 306209 (238249,371470) | 146491 (124841,173155) | 39.87 (34.3,45.96) | 54.57 (42.86,65.89) | 25.63 (21.91,30.24) | 834461 (673685,1015061) | 614373 (467869,792967) | 220088 (182603,268202) | 30.06 (24.25,36.54) | 45.97 (35.2,59.3) | 15.44 (12.8,18.77) |
| Low-middle SDI | 88349 (76931,106573) | 55155 (45623,72140) | 33194 (29168,36622) | 13.06 (11.48,15.88) | 16.21 (13.52,21.28) | 9.8 (8.65,10.77) | 157615 (139220,179612) | 98245 (81491,118824) | 59370 (52890,65650) | 10.34 (9.11,11.79) | 13.4 (11.1,16.15) | 7.51 (6.71,8.27) |
| Low SDI | 35766 (28501,40942) | 20576 (14768,24271) | 15190 (12362,17722) | 14.15 (11.27,16.18) | 16.37 (11.84,19.26) | 11.83 (9.72,13.82) | 57598 (45194,65610) | 32143 (22279,38000) | 25455 (20852,28901) | 10.22 (8.07,11.56) | 11.9 (8.29,14.01) | 8.61 (7.12,9.76) |
| Asia | 1149058 (1006236,1263723) | 780871 (646035,896007) | 368188 (329102,412593) | 53.34 (47.38,58.53) | 73.16 (61.64,83.78) | 34.12 (30.47,38.08) | 1834798 (1524826,2212979) | 1347252 (1070082,1702694) | 487546 (414688,573052) | 35.79 (29.87,42.98) | 54.47 (43.42,68.25) | 18.54 (15.7,21.77) |
| China | 615217 (503482,720422) | 431491 (319789,537802) | 183726 (148406,226732) | 67.17 (55.35,78.41) | 94.77 (70.95,117.86) | 40.33 (32.86,49.41) | 1226056 (943897,1546818) | 937643 (683807,1240047) | 288412 (223730,365255) | 57.22 (44.18,71.99) | 89.25 (65.15,117.49) | 26.71 (20.68,33.89) |
| Japan | 332600 (318214,341973) | 220805 (213912,227424) | 111795 (104112,116316) | 194.37 (185.97,199.86) | 287.13 (278.17,296.11) | 119.99 (112.16,124.62) | 265101 (234893,282114) | 183189 (168166,192774) | 81912 (65388,91659) | 78.91 (72.7,82.58) | 118.37 (110.86,123.33) | 44.88 (39.1,48.23) |
| South Korea | 44686 (32414,50798) | 29045 (19287,33841) | 15642 (11890,18818) | 130.74 (98.17,148.67) | 196.67 (135.84,230.01) | 82.33 (64.06,99.39) | 72258 (60323,92064) | 50257 (40560,68566) | 22001 (16965,27628) | 79.38 (66.67,100.81) | 118.13 (94.76,160.79) | 47.13 (36.97,58.66) |
| North Korea | 6763 (4882,8931) | 4265 (2968,6035) | 2498 (1807,3432) | 37.94 (27.73,49.55) | 57.8 (41.12,81.03) | 24.61 (17.86,33.74) | 12778 (9380,16340) | 8694 (6129,12068) | 4084 (2882,5664) | 37.47 (27.68,47.79) | 56.31 (40.1,78.5) | 21.89 (15.32,30.22) |
| Mongolia | 746 (603,934) | 437 (319,602) | 309 (231,416) | 66.68 (54.01,83.3) | 85.5 (63.16,116.66) | 51.25 (37.82,69.9) | 1196 (948,1521) | 825 (606,1113) | 371 (270,489) | 47.15 (37.79,58.6) | 72.67 (54.11,96.75) | 27.64 (20,36.43) |

**Table S2.** **Incidence of gastric cancer between 1990 and 2021 at the global, regional and 5 East-Asian countries level.**

| Location | 1990 Incident cases (95% UI) |  |  | 1990 Age-standardized rates per 100 000 people (95% UI) |  |  | 2021 Incident cases (95% UI) |  |  | 2021 Age-standardized rates per 100 000 people (95% UI) |  |  |
| --- | --- | --- | --- | --- | --- | --- | --- | --- | --- | --- | --- | --- |
|  | Total | Male | Female | Total | Male | Female | Total | Male | Female | Total | Male | Female |
| Global | 980899 (891307,1072236) | 628158 (540733,705759) | 352742 (321554,384689) | 24.76 (22.58,27) | 34.37 (30.04,38.67) | 16.61 (15.11,18.11) | 1230233 (1052350,1409970) | 832921 (687723,1006469) | 397312 (344272,446653) | 14.33 (12.23,16.41) | 20.94 (17.22,25.19) | 8.63 (7.47,9.7) |
| SDI |  | | | | | | | | | | | |
| High SDI | 254259 (242222,261672) | 157795 (151055,162925) | 96464 (88694,100635) | 23.13 (22.05,23.8) | 33.84 (32.29,34.94) | 15.14 (14,15.76) | 239119 (215071,256938) | 155312 (143697,167390) | 83807 (69422,92292) | 11.16 (10.21,11.91) | 16.25 (15.05,17.52) | 6.92 (5.98,7.51) |
| High-middle SDI | 332614 (298475,360843) | 214511 (180117,241881) | 118103 (107412,129981) | 33.33 (30.09,36.1) | 49.26 (41.7,55.21) | 21.1 (19.17,23.24) | 387196 (315957,457286) | 269803 (208738,335038) | 117393 (99387,137147) | 19.62 (16.02,23.13) | 30.25 (23.4,37.38) | 10.83 (9.17,12.68) |
| Middle SDI | 303889 (262410,351115) | 200344 (157082,242089) | 103545 (88663,122050) | 28.89 (25.13,33.44) | 38.97 (31.02,47.14) | 19.46 (16.77,22.86) | 451465 (368224,542292) | 315930 (244637,403226) | 135535 (113282,162089) | 16.91 (13.79,20.28) | 25.03 (19.36,31.7) | 9.74 (8.11,11.59) |
| Low-middle SDI | 63086 (55336,76805) | 39667 (33015,52148) | 23419 (20611,25782) | 10.14 (8.98,12.42) | 12.61 (10.54,16.58) | 7.6 (6.69,8.37) | 110396 (97098,126222) | 68245 (56334,82627) | 42151 (37681,46437) | 7.68 (6.71,8.77) | 9.93 (8.16,11.98) | 5.64 (5.05,6.2) |
| Low SDI | 26334 (20975,30114) | 15395 (11175,18127) | 10939 (8978,12790) | 11.41 (9.04,13.03) | 13.35 (9.75,15.88) | 9.41 (7.77,11) | 41388 (32600,46956) | 23213 (16190,27381) | 18176 (15013,20624) | 8.13 (6.44,9.22) | 9.5 (6.65,11.17) | 6.85 (5.68,7.75) |
| Asia | 645662 (559936,727668) | 430809 (348233,506348) | 214853 (188267,245120) | 31.86 (28.02,36.01) | 43.34 (35.68,50.92) | 21.07 (18.47,23.94) | 913055 (751620,1089660) | 641083 (504779,809415) | 271972 (226455,319744) | 18.3 (15.05,21.65) | 27.16 (21.28,34.02) | 10.45 (8.64,12.27) |
| China | 407471 (337565,477569) | 278596 (208188,346574) | 128875 (105159,157699) | 48.03 (40.21,56.69) | 67.64 (51.71,83.67) | 30.22 (24.8,36.89) | 611799 (471966,765562) | 446434 (325932,589284) | 165365 (127716,208140) | 29.05 (22.42,36.2) | 44.48 (32.18,58.38) | 15.23 (11.77,19.16) |
| Japan | 108286 (103064,111463) | 69791 (67357,71720) | 38495 (35290,40203) | 64.05 (60.74,66) | 94.85 (91.32,97.59) | 40.9 (37.65,42.67) | 99035 (85266,106694) | 65357 (59081,68825) | 33678 (25582,38297) | 25.54 (23.04,26.96) | 38.77 (35.83,40.51) | 14.66 (12.25,15.99) |
| South Korea | 22167 (16777,25107) | 14133 (9549,16266) | 8034 (6285,9666) | 71.18 (55.99,80.71) | 109.56 (78.14,128.02) | 45.63 (36.34,54.94) | 23664 (19710,29807) | 15910 (12954,21518) | 7754 (5757,9913) | 25.76 (21.53,32.4) | 38.98 (31.66,52.47) | 15.57 (11.94,19.69) |
| North Korea | 4622 (3365,6013) | 2807 (1968,3935) | 1815 (1312,2480) | 27.89 (20.62,35.8) | 42.51 (30.58,59.14) | 18.8 (13.8,25.38) | 8050 (6046,10116) | 5225 (3732,7327) | 2825 (2016,3939) | 24.02 (18.19,30.16) | 36.05 (25.84,50.83) | 14.85 (10.59,20.64) |
| Mongolia | 574 (466,715) | 338 (250,461) | 236 (174,320) | 54.22 (44.05,67.94) | 71.82 (53.56,97.07) | 40.68 (29.43,55.53) | 848 (680,1062) | 576 (422,773) | 272 (197,360) | 36.83 (29.39,45.27) | 56.73 (41.71,74.56) | 22.2 (15.92,29.51) |

**Table S3. Deaths of gastric cancer between 1990 and 2021 at the global, regional and 5 East-Asian countries level.**

| Location | 1990 Deaths cases (95% UI) |  |  | 1990 Age-standardized rates per 100 000 people (95% UI) |  |  | 2021 Deaths cases (95% UI) |  |  | 2021 Age-standardized rates per 100 000 people (95% UI) |  |  |
| --- | --- | --- | --- | --- | --- | --- | --- | --- | --- | --- | --- | --- |
|  | Total | Male | Female | Total | Male | Female | Total | Male | Female | Total | Male | Female |
| Global | 854185 (772885,939973) | 538665 (459835,612038) | 315520 (286766,345466) | 22.01 (20.03,24.19) | 30.41 (26.42,34.48) | 15.05 (13.6,16.46) | 954374 (821751,1089577) | 624551 (514019,750101) | 329822 (287083,368393) | 11.2 (9.62,12.73) | 16.03 (13.24,19.12) | 7.13 (6.22,7.97) |
| SDI |  | | | | | | | | | | | |
| High SDI | 175683 (166255,181197) | 105400 (100391,108963) | 70282 (64075,73637) | 15.86 (15.01,16.37) | 23.15 (22,23.93) | 10.69 (9.78,11.19) | 153539 (136670,165354) | 95941 (88356,103211) | 57598 (46895,63810) | 6.83 (6.18,7.34) | 9.9 (9.13,10.68) | 4.37 (3.71,4.78) |
| High-middle SDI | 304773 (274911,330960) | 194148 (163114,219408) | 110625 (100616,121960) | 31.08 (28.09,33.7) | 46.11 (39.12,51.67) | 19.92 (18.1,21.98) | 295105 (244787,343611) | 198606 (155929,244117) | 96499 (81769,112005) | 14.93 (12.4,17.36) | 22.73 (17.82,27.73) | 8.72 (7.43,10.12) |
| Middle SDI | 284016 (246760,329474) | 183953 (144299,223505) | 100064 (86464,117991) | 28.1 (24.61,32.65) | 37.48 (30.01,45.8) | 19.49 (16.92,22.9) | 356459 (294160,423770) | 240743 (189614,303926) | 115717 (97818,136377) | 13.72 (11.31,16.22) | 19.83 (15.59,24.81) | 8.45 (7.07,9.95) |
| Low-middle SDI | 62598 (54993,76278) | 39276 (32669,51637) | 23322 (20538,25666) | 10.45 (9.25,12.79) | 12.95 (10.82,17.01) | 7.88 (6.92,8.69) | 107456 (94064,122211) | 65856 (53708,79403) | 41600 (37227,45808) | 7.71 (6.69,8.76) | 9.92 (8.05,11.89) | 5.73 (5.12,6.29) |
| Low SDI | 26410 (21016,30213) | 15455 (11294,18207) | 10955 (9053,12857) | 11.9 (9.41,13.57) | 13.94 (10.2,16.61) | 9.82 (8.13,11.48) | 41192 (32515,46864) | 23024 (16146,27090) | 18168 (15001,20610) | 8.46 (6.71,9.6) | 9.87 (6.92,11.6) | 7.15 (5.93,8.12) |
| Asia | 551130 (475566,630497) | 362870 (287132,430163) | 188259 (163452,216348) | 28.11 (24.6,32.27) | 37.95 (30.69,45.2) | 19.02 (16.54,21.82) | 682477 (561131,809426) | 464020 (363368,586430) | 218457 (182816,255806) | 13.93 (11.47,16.44) | 20.29 (15.98,25.32) | 8.45 (7,9.89) |
| China | 374066 (310921,442251) | 251602 (188204,314409) | 122464 (100701,149718) | 46.05 (38.88,54.43) | 64.67 (49.6,79.93) | 29.81 (24.69,36.43) | 445013 (344736,555834) | 314779 (230725,418722) | 130234 (100509,163561) | 21.51 (16.66,26.61) | 32.61 (23.61,42.8) | 12.02 (9.29,15.1) |
| Japan | 56091 (52923,57893) | 35120 (33868,36186) | 20972 (18960,22001) | 33.76 (31.64,34.94) | 50.13 (48.01,51.79) | 22.19 (20.07,23.28) | 58012 (48988,63019) | 36673 (33103,38686) | 21339 (15775,24551) | 13.2 (11.67,14.04) | 20.26 (18.58,21.26) | 7.64 (6.23,8.45) |
| South Korea | 16261 (12515,18306) | 10196 (6968,11715) | 6065 (4857,7269) | 55.44 (44.07,62.45) | 86.29 (62.78,102.05) | 36.2 (29.28,43.6) | 12270 (10052,15312) | 7993 (6501,10786) | 4278 (3143,5545) | 13.26 (10.91,16.49) | 20.5 (16.51,27.32) | 8.08 (6.03,10.28) |
| North Korea | 4428 (3195,5749) | 2627 (1834,3637) | 1801 (1308,2451) | 27.76 (20.49,35.6) | 42.14 (30.3,58.33) | 19.16 (14.02,25.65) | 7135 (5391,9040) | 4494 (3235,6221) | 2641 (1890,3689) | 21.57 (16.41,27.08) | 32.32 (23.55,44.99) | 13.79 (9.82,19.28) |
| Mongolia | 585 (476,734) | 344 (255,470) | 241 (176,326) | 56.43 (45.75,71.23) | 75.52 (56.23,101.28) | 42.13 (30.29,57.27) | 825 (659,1028) | 554 (407,739) | 271 (195,361) | 37.4 (29.36,45.86) | 57.35 (42.44,75.28) | 22.99 (16.43,30.72) |

**Table S4.** **YLDs (Years Lived with Disability) of gastric cancer between 1990 and 2021 at the global, regional and 5 East-Asian countries level.**

| Location | 1990 YLDs cases (95% UI) |  |  | 1990 Age-standardized rates per 100 000 people (95% UI) |  |  | 2021 YLDs cases (95% UI) |  |  | 2021 Age-standardized rates per 100 000 people (95% UI) |  |  |
| --- | --- | --- | --- | --- | --- | --- | --- | --- | --- | --- | --- | --- |
|  | Total | Male | Female | Total | Male | Female | Total | Male | Female | Total | Male | Female |
| Global | 247909 (178784,324539) | 160829 (112259,210449) | 87080 (62341,114095) | 6.14 (4.44,8.01) | 8.55 (6,11.16) | 4.06 (2.91,5.3) | 325724 (232731,425362) | 224434 (155354,298433) | 101290 (71824,135415) | 3.77 (2.7,4.92) | 5.55 (3.85,7.36) | 2.21 (1.57,2.95) |
| SDI |  | | | | | | | | | | | |
| High SDI | 71148 (52634,90488) | 45216 (33458,58094) | 25932 (18859,32760) | 6.53 (4.84,8.32) | 9.56 (7.09,12.28) | 4.18 (3.05,5.29) | 68488 (50364,87060) | 45491 (33985,58199) | 22997 (16440,29698) | 3.31 (2.43,4.25) | 4.83 (3.6,6.19) | 2.03 (1.45,2.63) |
| High-middle SDI | 80287 (57134,104602) | 52432 (36236,70361) | 27855 (19828,36462) | 7.94 (5.65,10.32) | 11.7 (8.08,15.63) | 4.97 (3.54,6.51) | 101632 (70442,135888) | 72226 (48357,98532) | 29406 (20274,40160) | 5.17 (3.58,6.91) | 8.01 (5.39,10.88) | 2.76 (1.91,3.78) |
| Middle SDI | 74258 (51520,97753) | 49602 (32862,68529) | 24655 (16984,32594) | 6.82 (4.75,8.92) | 9.27 (6.18,12.82) | 4.49 (3.13,5.94) | 117669 (81178,158485) | 83899 (54388,115694) | 33769 (23073,45727) | 4.32 (3,5.81) | 6.47 (4.2,8.91) | 2.4 (1.64,3.24) |
| Low-middle SDI | 15618 (11055,20888) | 9737 (6529,13906) | 5881 (4088,7830) | 2.42 (1.74,3.25) | 2.99 (2.01,4.26) | 1.84 (1.28,2.44) | 27504 (19162,36538) | 16960 (11499,23164) | 10544 (7363,13823) | 1.87 (1.31,2.48) | 2.4 (1.63,3.26) | 1.38 (0.96,1.81) |
| Low SDI | 6428 (4446,8552) | 3735 (2313,5224) | 2693 (1841,3625) | 2.68 (1.84,3.54) | 3.12 (1.93,4.33) | 2.22 (1.54,3) | 10264 (6889,13602) | 5752 (3686,7945) | 4512 (3119,6019) | 1.93 (1.31,2.56) | 2.25 (1.44,3.12) | 1.63 (1.12,2.16) |
| Asia | 166200 (118234,219445) | 112110 (76100,150450) | 54090 (38222,71312) | 7.95 (5.68,10.45) | 10.88 (7.43,14.6) | 5.15 (3.64,6.75) | 243769 (168256,325575) | 174318 (117374,237577) | 69450 (47754,94985) | 4.82 (3.35,6.4) | 7.21 (4.87,9.77) | 2.65 (1.83,3.62) |
| China | 99457 (67257,133581) | 68987 (44150,96944) | 30469 (20726,41516) | 11.32 (7.68,15.1) | 16.02 (10.24,22.33) | 6.94 (4.71,9.4) | 162261 (108718,224613) | 120905 (75513,171020) | 41357 (26669,58573) | 7.64 (5.13,10.57) | 11.79 (7.37,16.54) | 3.82 (2.46,5.42) |
| Japan | 33920 (25554,43526) | 22336 (16832,28983) | 11584 (8697,14755) | 19.95 (15.01,25.58) | 29.63 (22.41,38.28) | 12.41 (9.34,15.79) | 28635 (21209,36962) | 19441 (14483,24954) | 9194 (6485,12192) | 8.04 (6.01,10.47) | 12.14 (9.01,15.7) | 4.58 (3.28,6.08) |
| South Korea | 5903 (3953,7678) | 3800 (2407,5050) | 2103 (1370,2818) | 18.07 (12.48,23.27) | 27.49 (18.44,36.82) | 11.49 (7.61,15.46) | 7378 (4994,10288) | 5045 (3384,7350) | 2333 (1521,3361) | 8.09 (5.46,11.31) | 12.09 (8.12,17.5) | 4.88 (3.2,6.93) |
| North Korea | 1134 (713,1620) | 697 (412,1037) | 437 (266,633) | 6.61 (4.22,9.33) | 10.04 (6.2,14.76) | 4.41 (2.71,6.35) | 2025 (1274,3061) | 1338 (777,2138) | 687 (415,1056) | 5.99 (3.83,8.99) | 8.96 (5.35,14.26) | 3.64 (2.21,5.58) |
| Mongolia | 133 (90,187) | 79 (51,117) | 54 (33,82) | 12.26 (8.36,17.42) | 16.12 (10.6,23.74) | 9.22 (5.62,14.06) | 202 (134,279) | 138 (89,205) | 64 (41,93) | 8.4 (5.65,11.57) | 12.95 (8.55,18.46) | 5 (3.19,7.25) |

**Table S5. YLLs (Years of Life Lost) of gastric cancer between 1990 and 2021 at the global, regional and 5 East-Asian countries level.**

| Location | 1990 YLLs cases (95% UI) |  |  | 1990 Age-standardized rates per 100 000 people (95% UI) |  |  | 2021 YLLs cases (95% UI) |  |  | 2021 Age-standardized rates per 100 000 people (95% UI) |  |  |
| --- | --- | --- | --- | --- | --- | --- | --- | --- | --- | --- | --- | --- |
|  | Total | Male | Female | Total | Male | Female | Total | Male | Female | Total | Male | Female |
| Global | 22989383 (20384886,25277838) | 14921047 (12441011,16966449) | 8068336 (7341263,8876456) | 553.58 (493.58,609.4) | 759.14 (640.63,861) | 369.58 (336.58,406.56) | 22460909 (19337840,25781644) | 15049913 (12384637,18378230) | 7410997 (6607549,8283387) | 258.98 (223.19,297.13) | 365.69 (301.34,444) | 163.35 (145.88,182.67) |
| SDI |  | | | | | | | | | | | |
| High SDI | 4028274 (3842178,4146666) | 2530399 (2404594,2621101) | 1497875 (1390215,1558870) | 374.6 (356.71,385.52) | 532.36 (506.62,551.34) | 250.05 (234.19,259.96) | 2828916 (2600869,3026083) | 1854911 (1732668,2022218) | 974005 (844944,1055161) | 142.79 (132.68,152.63) | 201.86 (188.86,220.6) | 91.39 (82.33,98.01) |
| High-middle SDI | 8160951 (7211487,8921568) | 5418818 (4477724,6124967) | 2742133 (2474832,3043602) | 794.81 (704.75,867.95) | 1166.22 (969.96,1318.36) | 489.6 (441.86,543.74) | 6799975 (5631965,8040423) | 4750042 (3715114,5870435) | 2049934 (1761803,2389454) | 348.01 (288.25,411.61) | 523.43 (409.68,644.26) | 196.13 (168.64,228.75) |
| Middle SDI | 8134338 (6974137,9411468) | 5365918 (4153389,6496587) | 2768421 (2373245,3266647) | 714.99 (618.34,827.75) | 954.85 (746.17,1154.59) | 482.62 (415.06,567.85) | 8703048 (7243729,10425107) | 5998077 (4705872,7603719) | 2704972 (2295720,3184824) | 315.92 (262.53,377.98) | 453.61 (357.58,572.2) | 190.57 (162.17,224.01) |
| Low-middle SDI | 1859186 (1615642,2240863) | 1148262 (945303,1500625) | 710925 (623979,786083) | 272.03 (237.83,330.27) | 334.58 (277.2,439.31) | 207.04 (182.07,228.09) | 2925621 (2579101,3320725) | 1789891 (1473194,2172977) | 1135729 (1009487,1259339) | 190.69 (167.97,216.67) | 242.68 (199.01,293.69) | 142.57 (126.98,157.55) |
| Low SDI | 789212 (631384,903525) | 446585 (322094,526213) | 342628 (278823,399755) | 309.3 (246.56,354.12) | 352.7 (256.43,414.79) | 263.73 (217.22,309.08) | 1188639 (931985,1359881) | 647708 (451702,767023) | 540931 (441709,614884) | 207.84 (164.01,236.8) | 237.13 (166.13,279.33) | 179.96 (148.3,204.28) |
| Asia | 15571064 (13186742,17632552) | 10401229 (8113611,12418332) | 5169836 (4493070,5955063) | 706.51 (604.03,803.87) | 950.87 (750.56,1129.56) | 467.57 (406.53,538.03) | 16165336 (13496266,19298040) | 11222816 (8797719,14379477) | 4942520 (4218895,5796339) | 314.27 (262.16,374.44) | 451.42 (354.58,575.25) | 187.63 (160.43,219.6) |
| China | 10674000 (8779197,12534506) | 7330291 (5396599,9160778) | 3343709 (2708265,4119979) | 1170.29 (969.14,1379.01) | 1618.83 (1205.91,2022.86) | 736.2 (599.94,903.37) | 10479865 (8094751,13211220) | 7619454 (5551448,10208683) | 2860411 (2219304,3632563) | 493.62 (381.57,620.17) | 738.6 (543.72,982.8) | 265.02 (205.67,336.63) |
| Japan | 1298633 (1248476,1330214) | 829711 (806747,850925) | 468921 (438742,483662) | 770.81 (739.97,790.23) | 1099.31 (1064.87,1129.03) | 517.95 (486.87,533.2) | 896598 (793196,953646) | 600170 (555677,627800) | 296428 (236966,330571) | 262.13 (242.16,274.16) | 387.11 (364.19,402.59) | 155.75 (136.88,166.4) |
| South Korea | 483220 (352359,543426) | 304982 (201704,351166) | 178238 (138709,212702) | 1417.32 (1077.54,1595.82) | 2096.34 (1447.01,2419.99) | 935.43 (738.64,1113.37) | 252687 (211541,321232) | 171267 (140170,233424) | 81420 (62884,102626) | 280.73 (235.04,355.98) | 409.51 (334.96,554.66) | 177.06 (139.81,221.13) |
| North Korea | 131449 (93722,172855) | 81649 (55846,113618) | 49800 (35790,69012) | 733.12 (527.6,954.05) | 1098.78 (771.18,1526.01) | 488.19 (352.29,670.05) | 198012 (146522,253420) | 132350 (92961,181650) | 65662 (45770,92922) | 579.76 (432.8,740.84) | 855.84 (611.13,1179.61) | 354.04 (246.04,498.45) |
| Mongolia | 16317 (13138,20490) | 9592 (6955,13264) | 6725 (4975,9025) | 1450.66 (1173.94,1826.22) | 1863.26 (1366.97,2551.13) | 1111.74 (821.07,1504.57) | 23861 (18843,30287) | 16666 (12170,22561) | 7195 (5214,9553) | 922.05 (741.04,1148.01) | 1435.27 (1059.47,1904.84) | 527.05 (381.13,697.34) |

**Table S6.** **DALYs (Disability-Adjusted Life Years) of gastric cancer between 1990 and 2021 at the global, regional and 5 East-Asian countries level.**

| Location | 1990 DALYs cases (95% UI) |  |  | 1990 Age-standardized rates per 100 000 people (95% UI) |  |  | 2021 DALYs cases (95% UI) |  |  | 2021 Age-standardized rates per 100 000 people (95% UI) |  |  |
| --- | --- | --- | --- | --- | --- | --- | --- | --- | --- | --- | --- | --- |
|  | Total | Male | Female | Total | Male | Female | Total | Male | Female | Total | Male | Female |
| Global | 23237292 (20605349,25526194) | 15081876 (12580522,17166413) | 8155416 (7415209,8979484) | 559.72 (499.09,615.77) | 767.69 (648.14,872.01) | 373.64 (340.07,410.99) | 22786633 (19576344,26118869) | 15274347 (12568802,18643478) | 7512286 (6716178,8393835) | 262.75 (226.08,301.02) | 371.24 (305.91,451.15) | 165.57 (148.13,185.02) |
| SDI |  | | | | | | | | | | | |
| High SDI | 4099422 (3902053,4218897) | 2575615 (2447951,2666363) | 1523807 (1413966,1584690) | 381.13 (362.21,392.23) | 541.92 (515.8,560.88) | 254.23 (238.09,264.29) | 2897404 (2654584,3101177) | 1900402 (1776034,2072884) | 997002 (866070,1082810) | 146.1 (135.56,155.89) | 206.69 (193.83,226.22) | 93.41 (84.17,100.15) |
| High-middle SDI | 8241238 (7281203,9010822) | 5471251 (4517698,6197182) | 2769988 (2502341,3077788) | 802.75 (711.79,876.76) | 1177.92 (978.94,1332.53) | 494.57 (446.35,549.92) | 6901607 (5703610,8141888) | 4822268 (3764569,5962596) | 2079339 (1786082,2427980) | 353.18 (291.89,416.78) | 531.44 (415.47,654.14) | 198.9 (170.73,232.29) |
| Middle SDI | 8208596 (7034443,9506074) | 5415520 (4186369,6554892) | 2793076 (2395796,3296099) | 721.81 (624.06,835.38) | 964.12 (752.9,1166.21) | 487.1 (418.55,572.69) | 8820717 (7336337,10567624) | 6081976 (4762325,7706264) | 2738741 (2324820,3225338) | 320.24 (266.07,382.87) | 460.07 (362.03,579.99) | 192.96 (164.11,227.05) |
| Low-middle SDI | 1874805 (1629366,2261562) | 1157999 (954317,1514611) | 716806 (629585,792945) | 274.45 (239.89,333.49) | 337.57 (279.69,443.63) | 208.87 (183.77,229.87) | 2953125 (2599859,3357038) | 1806852 (1487676,2193456) | 1146274 (1017981,1271096) | 192.56 (169.42,219.14) | 245.08 (201.05,296.57) | 143.95 (128.12,159.09) |
| Low SDI | 795640 (636078,910967) | 450320 (324378,531063) | 345320 (281009,402619) | 311.98 (248.62,357.31) | 355.82 (258.35,418.27) | 265.95 (219.09,311.7) | 1198903 (940407,1371290) | 653460 (456045,774130) | 545443 (445244,619601) | 209.77 (165.6,238.95) | 239.38 (167.84,282.13) | 181.59 (149.67,206.26) |
| Asia | 15737264 (13322575,17823025) | 10513338 (8200689,12536735) | 5223925 (4537081,6022276) | 714.46 (610.59,812.06) | 961.75 (758.66,1144.27) | 472.72 (410.82,544.23) | 16409105 (13701719,19619475) | 11397134 (8934549,14600017) | 5011971 (4280452,5872139) | 319.08 (266.18,379.89) | 458.63 (360.63,583.92) | 190.28 (162.71,222.34) |
| China | 10773457 (8850977,12638919) | 7399279 (5450273,9252517) | 3374178 (2734453,4160646) | 1181.61 (978.38,1390.89) | 1634.85 (1218.61,2045.07) | 743.14 (605.54,913.28) | 10642127 (8222106,13383779) | 7740359 (5634331,10365104) | 2901768 (2251657,3679391) | 501.26 (387.29,627.98) | 750.39 (550.9,997.91) | 268.83 (208.91,340.98) |
| Japan | 1332552 (1283575,1367533) | 852047 (825707,873871) | 480505 (450000,496621) | 790.75 (759.64,811.78) | 1128.94 (1092.71,1158.55) | 530.36 (498.75,547.42) | 925233 (815855,984384) | 619611 (572456,648582) | 305622 (244335,340632) | 270.17 (248.93,282.24) | 399.25 (375.11,416.15) | 160.33 (141.01,171.15) |
| South Korea | 489123 (356246,549576) | 308782 (204534,355443) | 180341 (140399,215232) | 1435.39 (1091.49,1613.83) | 2123.84 (1468.42,2455.3) | 946.92 (749.11,1128.55) | 260065 (218481,330431) | 176312 (144122,239486) | 83753 (64960,105348) | 288.82 (242.93,366.07) | 421.6 (344.2,568.98) | 181.94 (143.4,227.76) |
| North Korea | 132583 (94428,174137) | 82346 (56345,114268) | 50237 (36138,69525) | 739.73 (531.88,961.88) | 1108.83 (777.92,1539.1) | 492.6 (355.36,676.41) | 200037 (148083,255936) | 133688 (93925,183687) | 66349 (46264,93854) | 585.75 (437.3,748.46) | 864.8 (617.32,1192.63) | 357.68 (248.26,503.33) |
| Mongolia | 16450 (13255,20644) | 9670 (7004,13372) | 6780 (5016,9115) | 1462.93 (1184.84,1839.72) | 1879.38 (1379.1,2572.74) | 1120.96 (827.55,1519.76) | 24064 (19036,30532) | 16804 (12282,22770) | 7259 (5263,9638) | 930.45 (747.52,1157.92) | 1448.22 (1070.03,1922.77) | 532.05 (384.75,703.8) |
