# Supplementary material for: Burden and Future Trends of Gastric Cancer in 5 East Asian Countries From 1990 to 2036: Epidemiological Study Analysis Using the Global Burden of Diseases Study 2021
Source: JMIR Cancer. 2025 Sep 3;11:e74389. doi: 10.2196/74389 (PMC12408060; doi:10.2196/74389)

Multimedia Appendix 2: Age-specific prevalence, incidence, death, YLDs, YLLs, and DALYs numbers in 5 East Asian countries.

Table of content

Figure S1. Age-specific prevalence numbers in 5 East Asian countries.

Figure S2. Age-specific incidence numbers in 5 East Asian countries.

Figure S3. Age-specific death numbers in 5 East Asian countries.

Figure S4. Age-specific YLDs numbers in 5 East Asian countries.

Figure S5. Age-specific YLLs numbers in 5 East Asian countries.

Figure S6. Age-specific DALYs numbers in 5 East Asian countries.

**Figure S1. Age-specific prevalence numbers in 5 East Asian countries.**


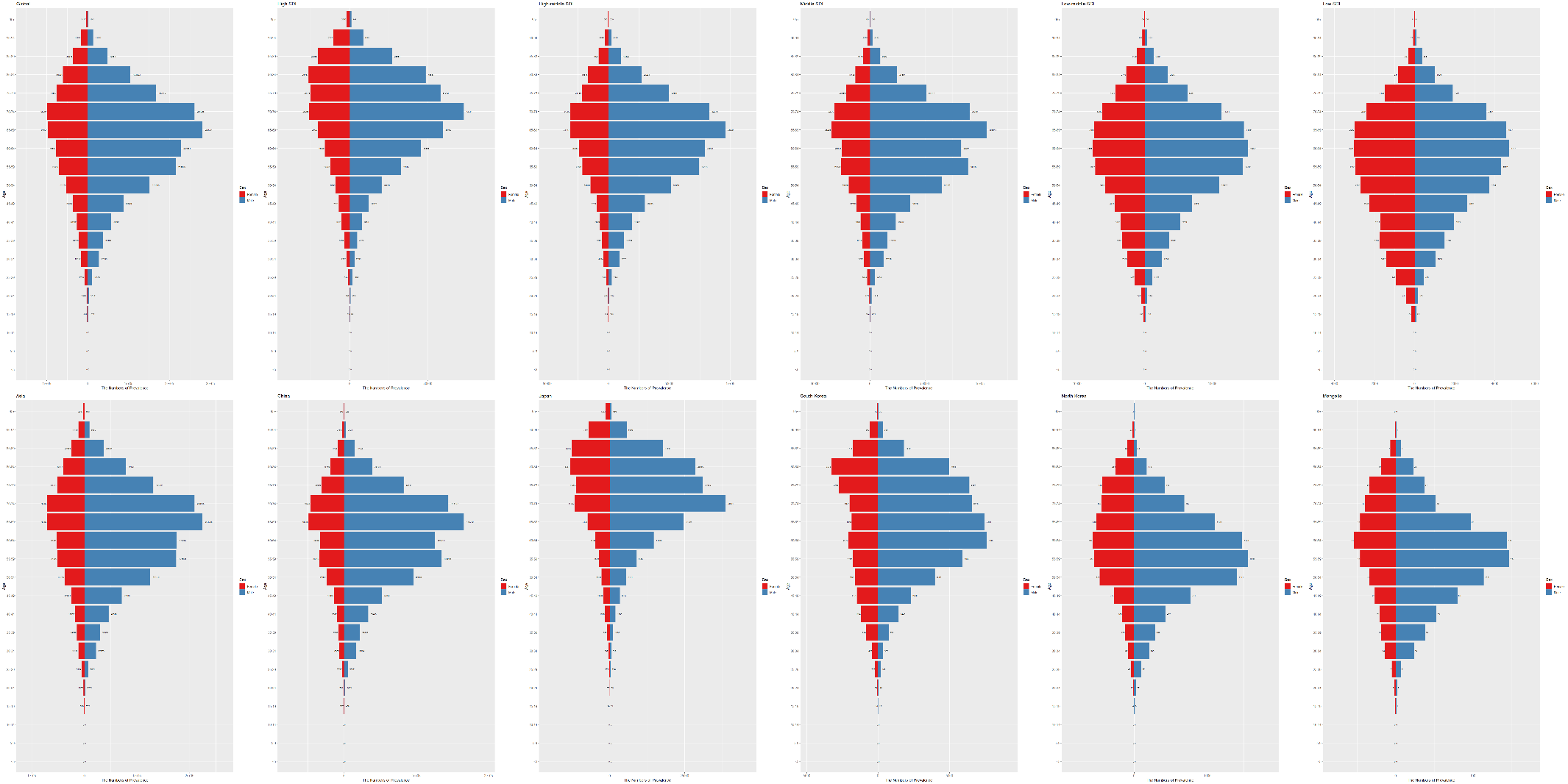


**Figure S2. Age-specific incidence numbers in 5 East Asian countries.**


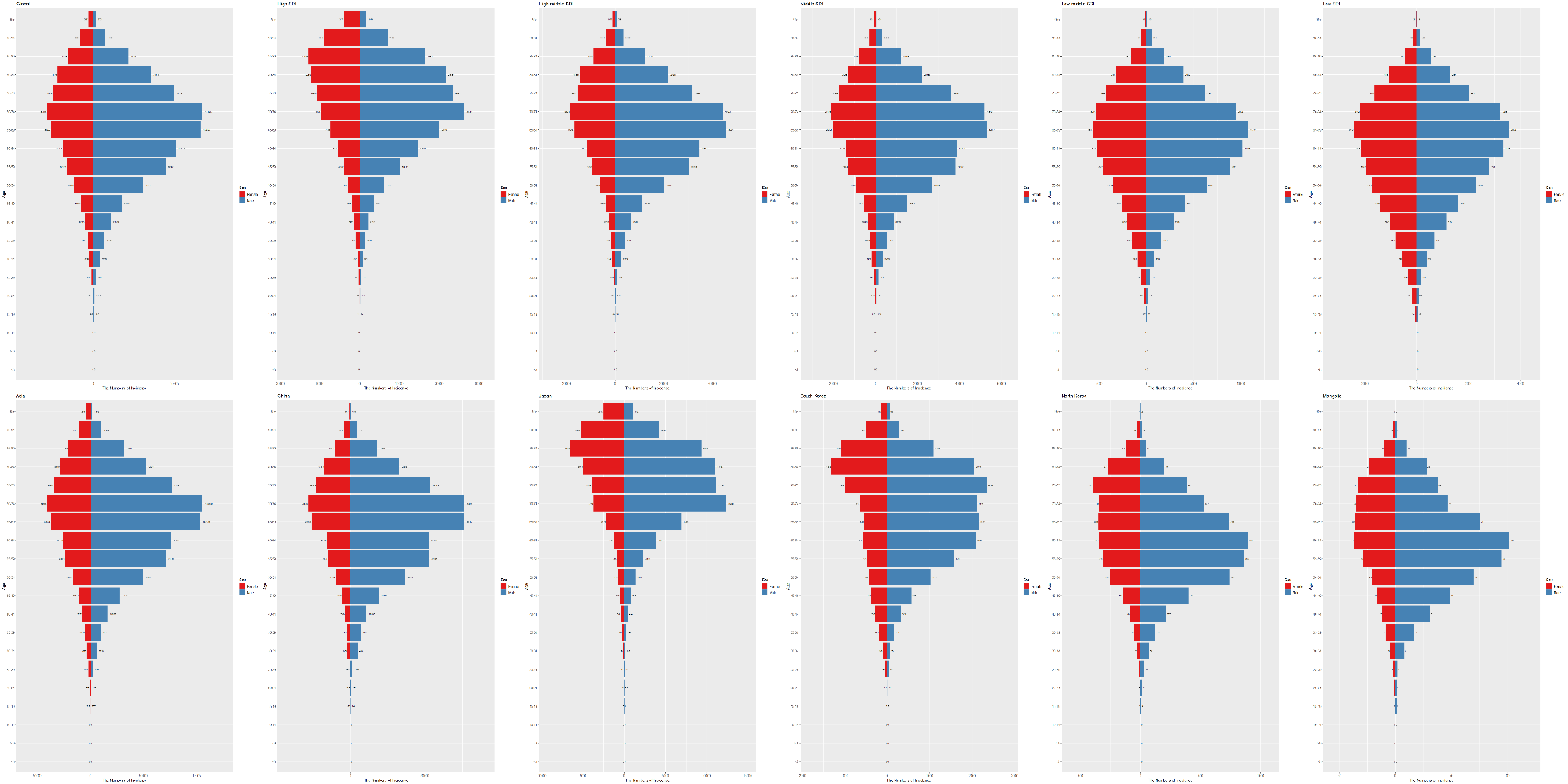


**Figure S3. Age-specific death numbers in 5 East Asian countries.**


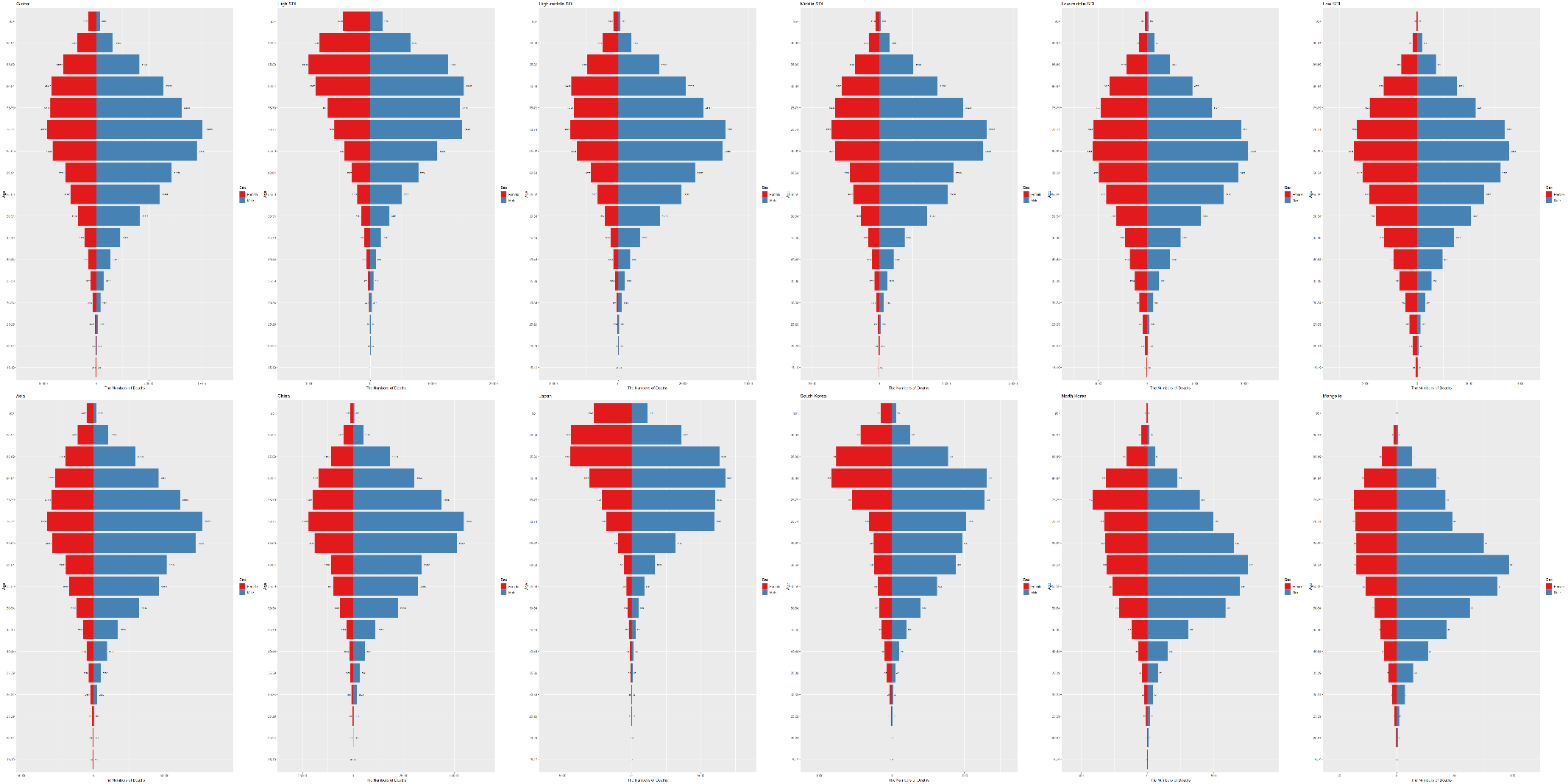


**Figure S4. Age-specific YLDs numbers in 5 East Asian countries.**


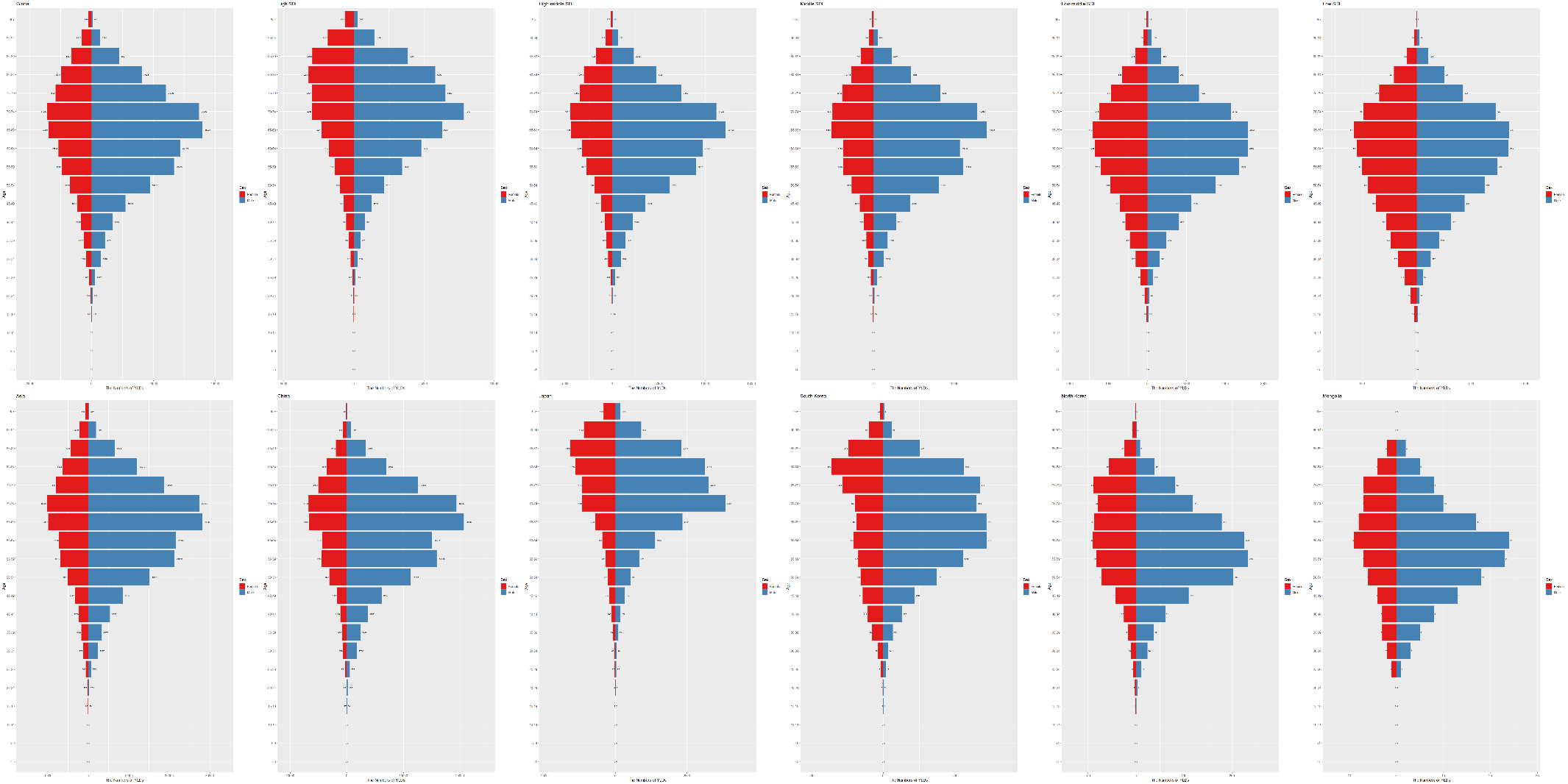


**Figure S5. Age-specific YLLs numbers in 5 East Asian countries.**


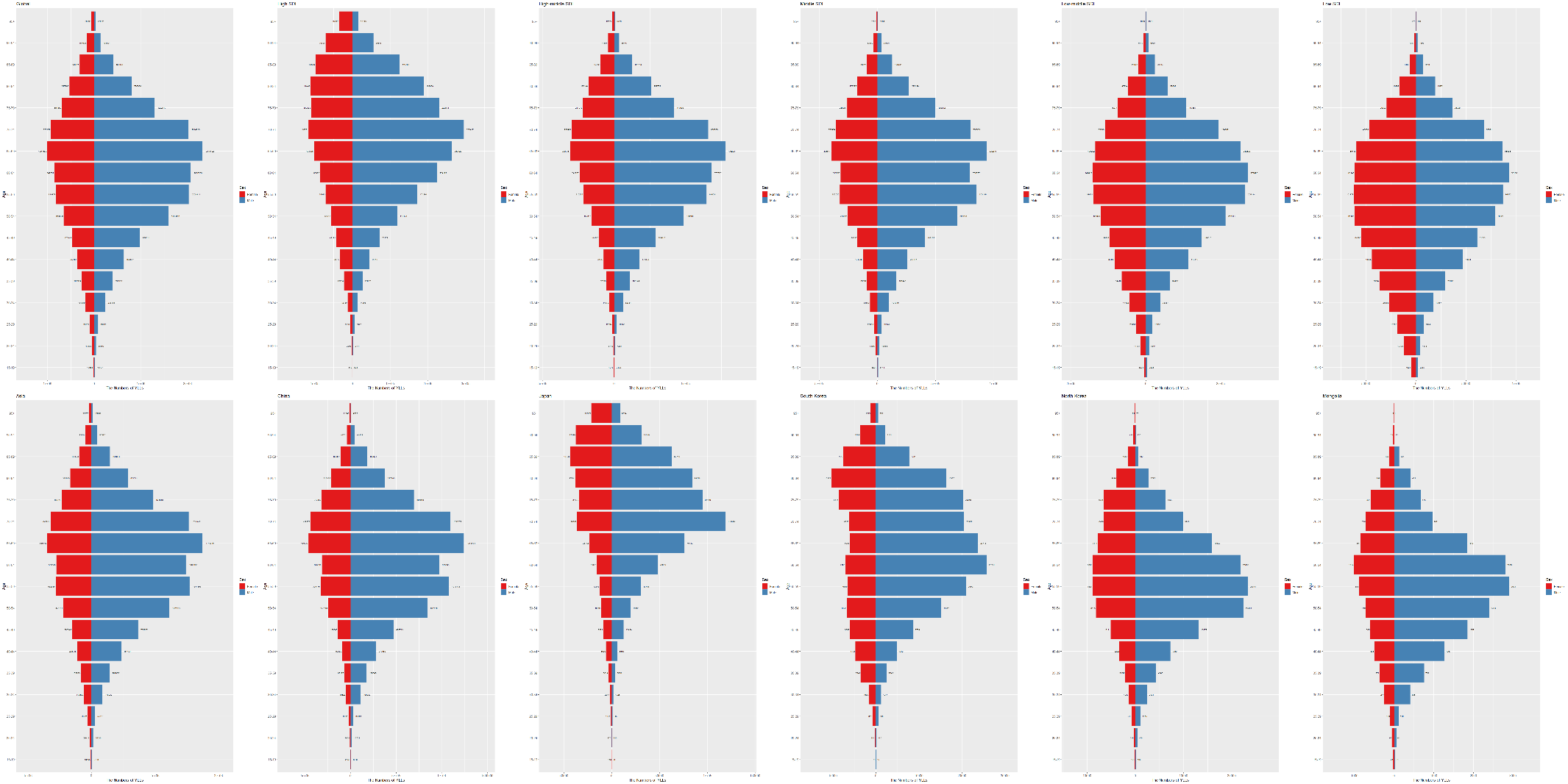


**Figure S6. Age-specific DALYs numbers in 5 East Asian countries.**


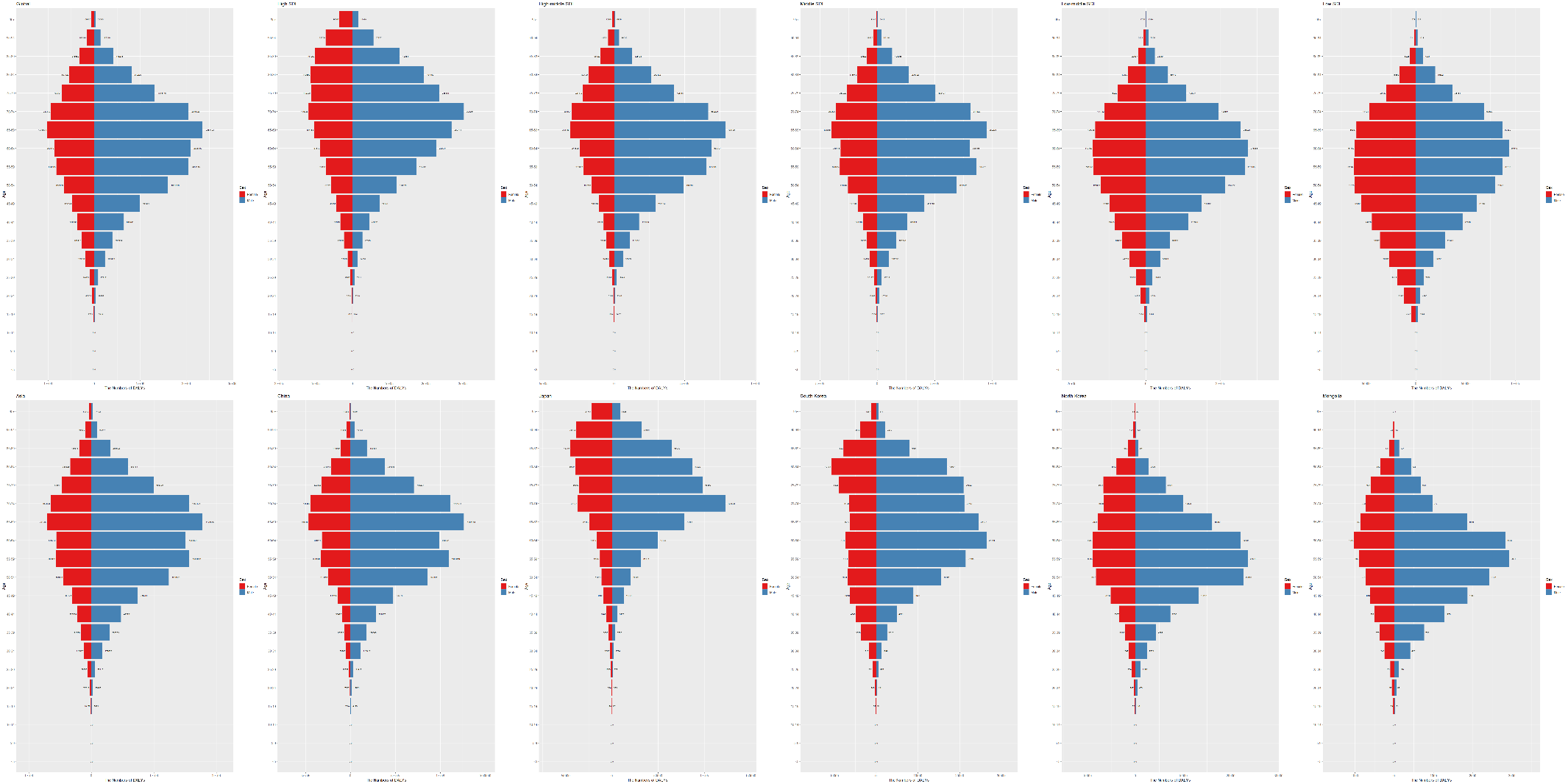

Supplement: Multimedia Appendix 2 [file cancer-v11-e74389-s002.docx]
