# Supplementary material for: Burden and Future Trends of Gastric Cancer in 5 East Asian Countries From 1990 to 2036: Epidemiological Study Analysis Using the Global Burden of Diseases Study 2021
Source: JMIR Cancer. 2025 Sep 3;11:e74389. doi: 10.2196/74389 (PMC12408060; doi:10.2196/74389)

Multimedia Appendix 3: Trends in the all-age prevalence, incidence, death, YLDs, YLLs, and DALYs number and rate by sex from 1990 to 2021.

Table of content

Figure S7. Trends in the all-age prevalence number and rate by sex from 1990 to 2021.

Figure S8. Trends in the all-age incidence number and rate by sex from 1990 to 2021.

Figure S9. Trends in the all-age death number and rate by sex from 1990 to 2021.

Figure S10. Trends in the all-age YLDs number and rate by sex from 1990 to 2021.

Figure S11. Trends in the all-age YLLs number and rate by sex from 1990 to 2021.

Figure S12. Trends in the all-age DALYs number and rate by sex from 1990 to 2021.

**Figure S7. Trends in the all-age prevalence number and rate by sex from 1990 to 2021.**


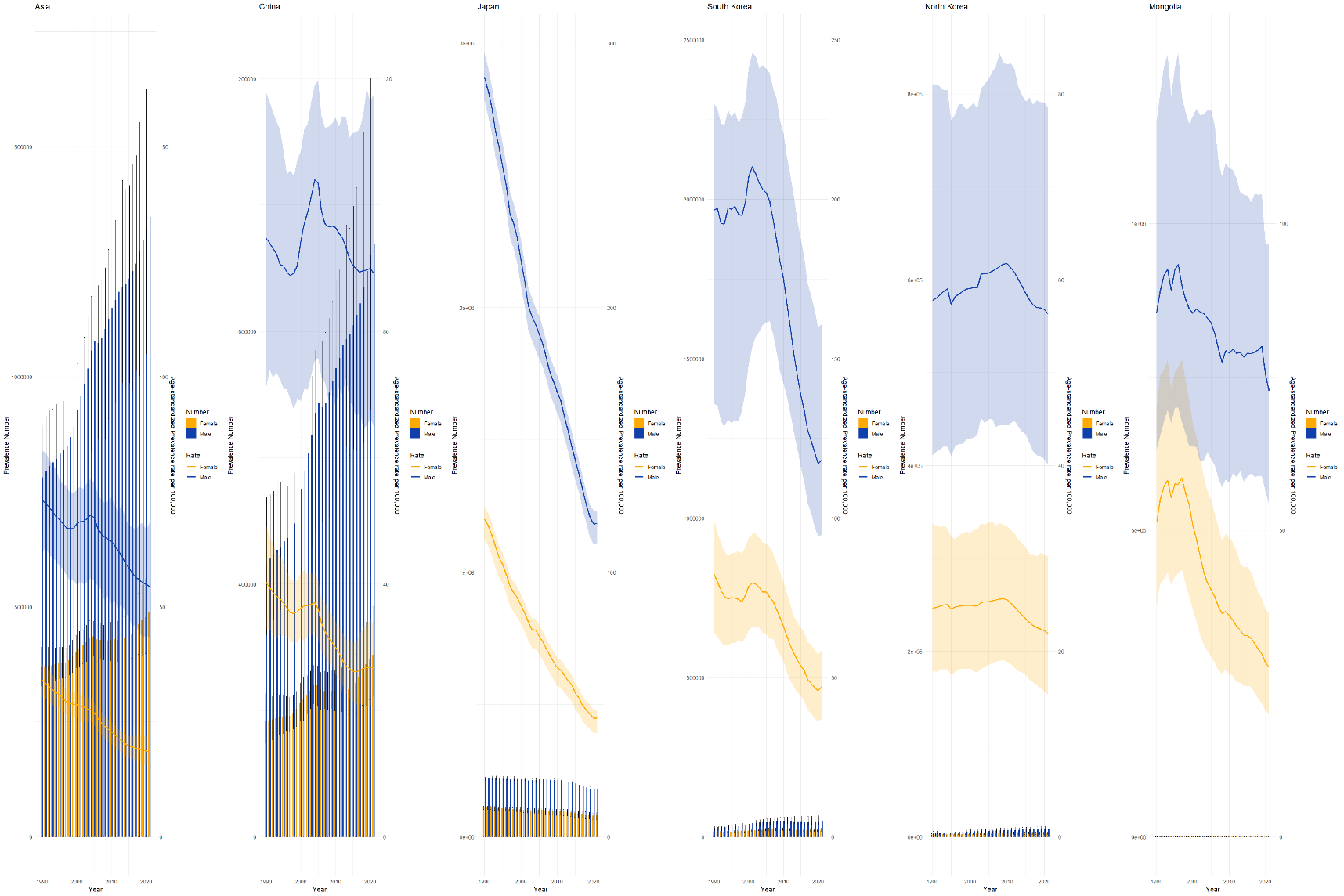


**Figure S8. Trends in the all-age incidence number and rate by sex from 1990 to 2021.**


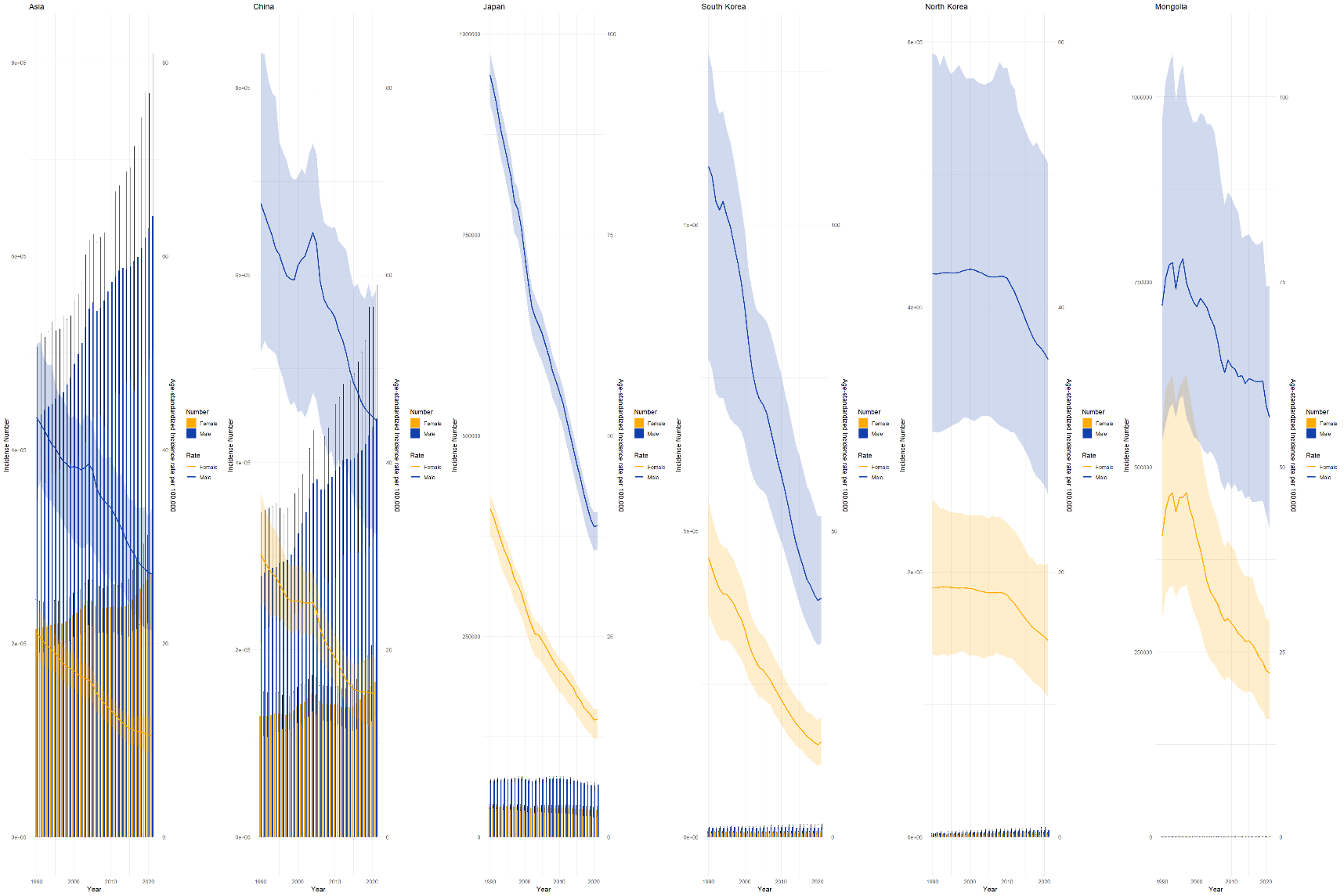


**Figure S9. Trends in the all-age death number and rate by sex from 1990 to 2021.**


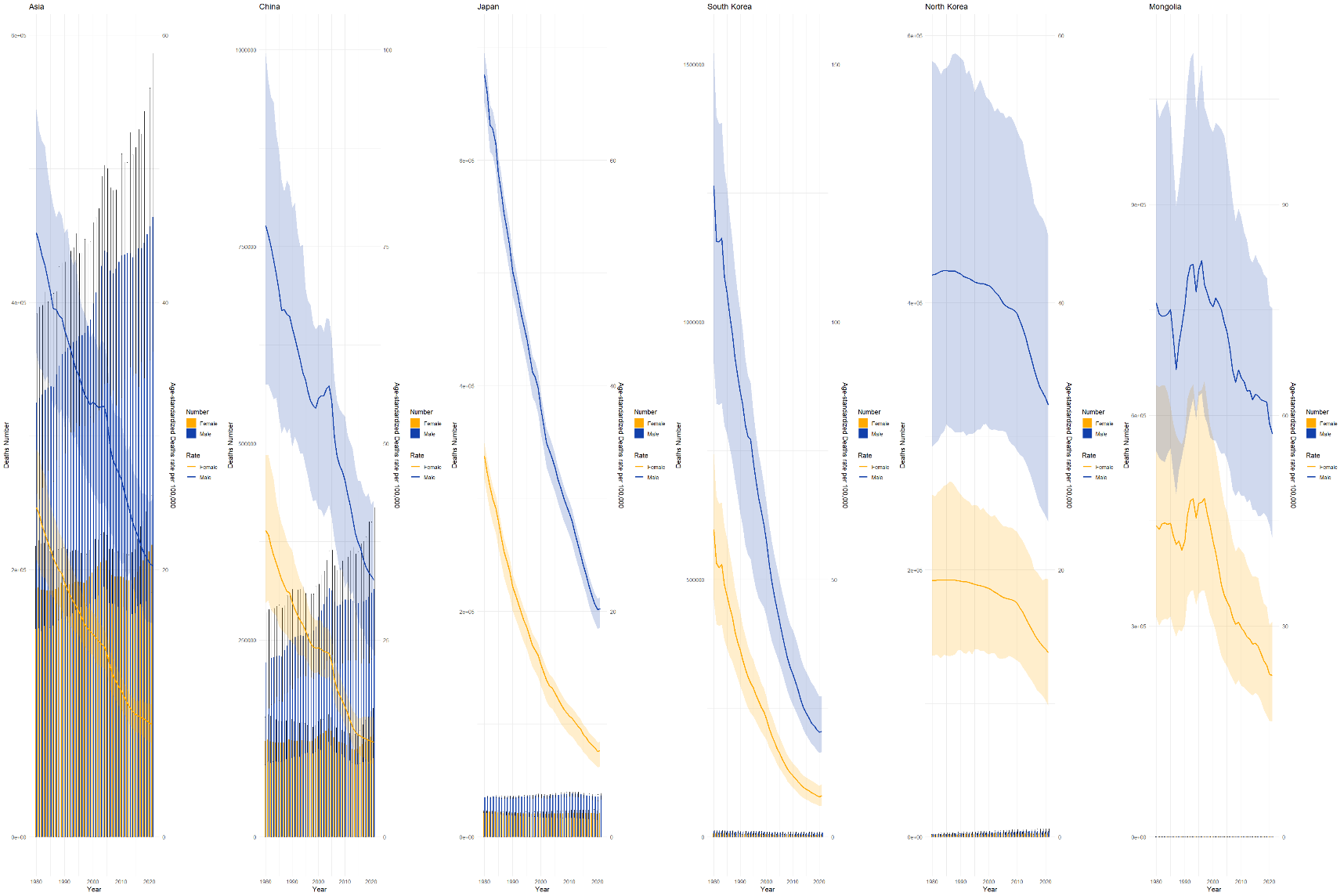


**Figure S10. Trends in the all-age YLDs number and rate by sex from 1990 to 2021.**


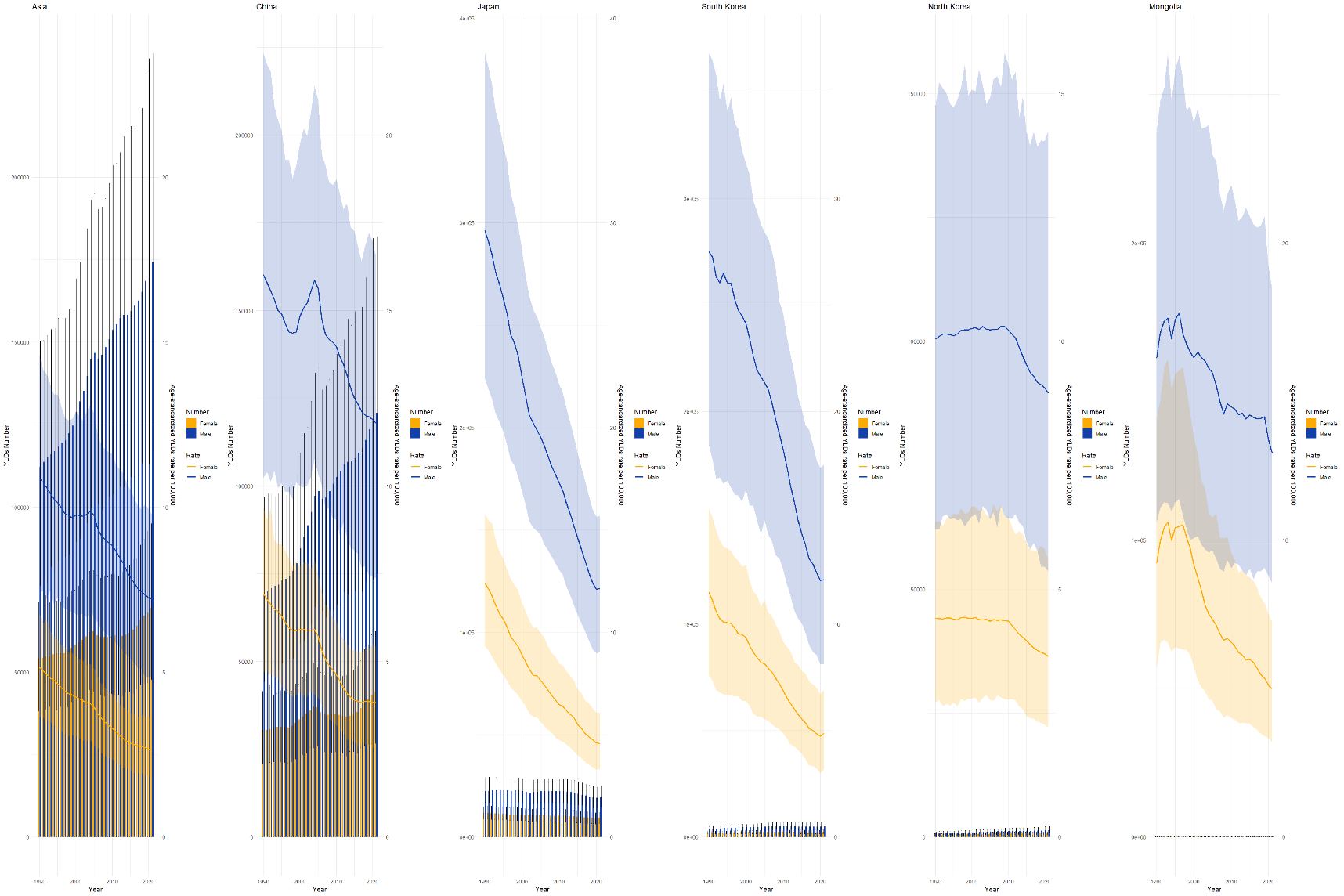


**Figure S11. Trends in the all-age YLLs number and rate by sex from 1990 to 2021.**


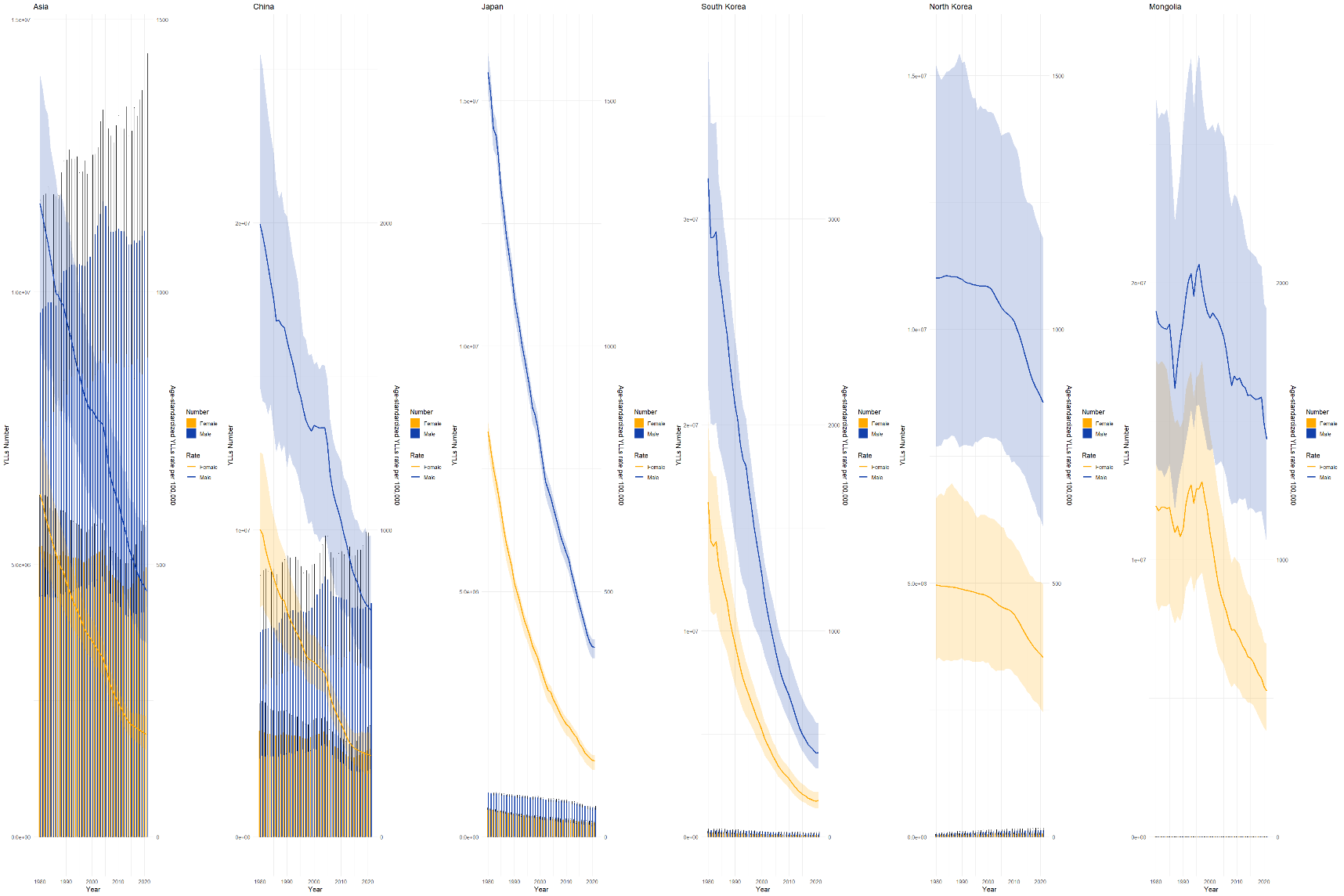


**Figure S12. Trends in the all-age DALYs number and rate by sex from 1990 to 2021.**


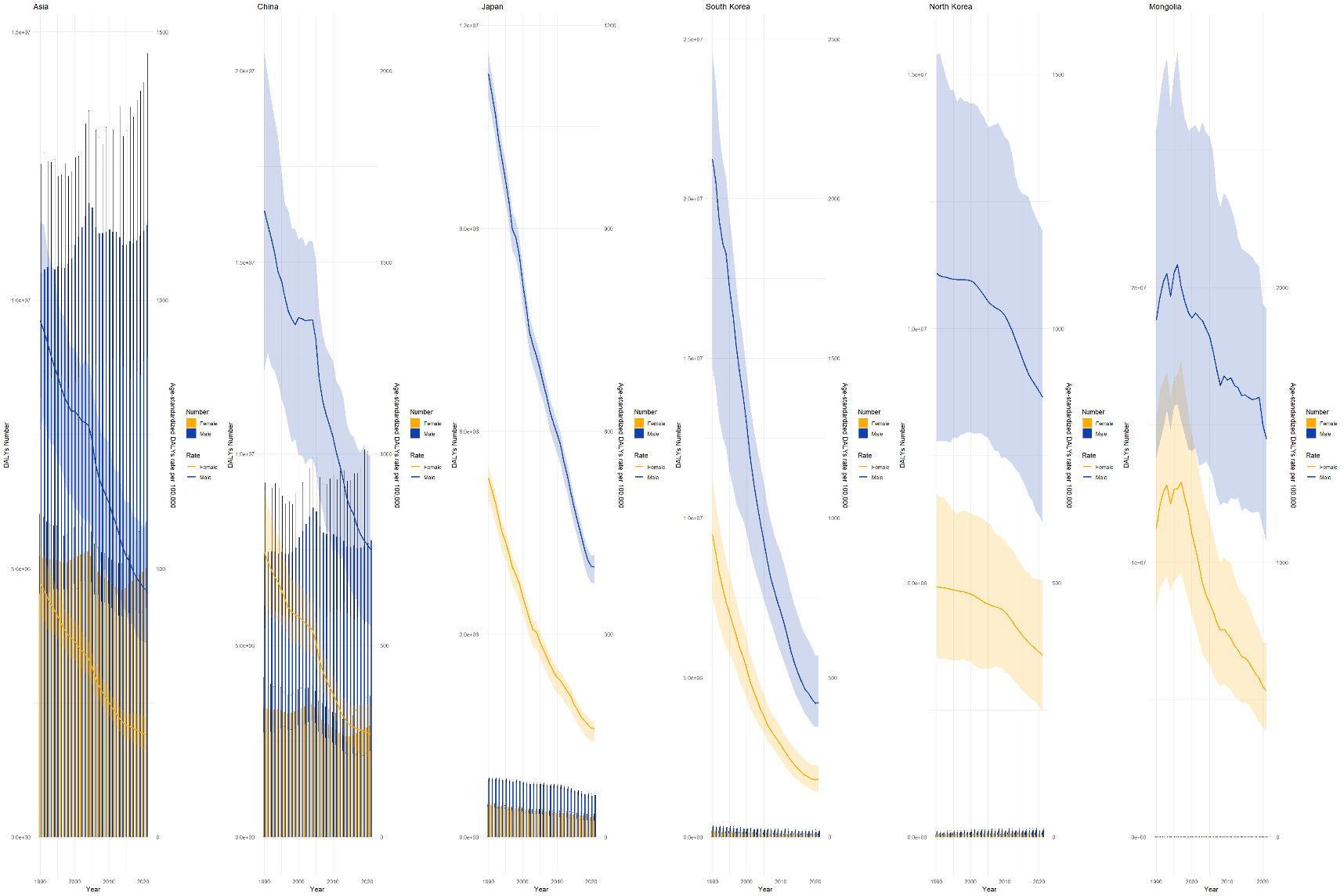

Supplement: Multimedia Appendix 3 [file cancer-v11-e74389-s003.docx]
