# Supplementary material for: Burden and Future Trends of Gastric Cancer in 5 East Asian Countries From 1990 to 2036: Epidemiological Study Analysis Using the Global Burden of Diseases Study 2021
Source: JMIR Cancer. 2025 Sep 3;11:e74389. doi: 10.2196/74389 (PMC12408060; doi:10.2196/74389)
Supplement: Multimedia Appendix 4 [file cancer-v11-e74389-s004.docx]

**Table S7.**

| Location | ASPR (95% CI) | ASIR (95% CI) | ASMR (95% CI) | Age-standardized YLDs rate (95% CI) | Age-standardized YLLs rate (95% CI) | ASDR (95% CI) |
| --- | --- | --- | --- | --- | --- | --- |
| Global | -1.26 (-1.40, -1.12) | -1.76 (-1.89, -1.64) | -2.11 (-2.27, -1.95) | -1.57 (-1.70, -1.44) | -2.36 (-2.55, -2.17) | -2.42 (-2.52, -2.33) |
| SDI |  |  |  |  |  |  |
| High SDI | -2.04 (-2.12, -1.96) | -2.34 (-2.48, -2.20) | -2.71 (-2.81, -2.61) | -2.18 (-2.27, -2.09) | -3.05 (-3.12, -2.98) | -3.07 (-3.15, -3.00) |
| High-middle SDI | -0.76 (-0.91, -0.61) | -1.69 (-1.84, -1.55) | -2.25 (-2.57, -1.93) | -1.38 (-1.50, -1.25) | -2.57 (-2.80, -2.35) | -2.62 (-2.80, -2.44) |
| Middle SDI | -0.91 (-1.02, -0.79) | -1.73 (-1.88, -1.59) | -2.17 (-2.43, -1.91) | -1.47 (-1.60, -1.33) | -2.46 (-2.65, -2.27) | -2.62 (-2.80, -2.45) |
| Low-middle SDI | -0.74 (-0.83, -0.65) | -0.87 (-1.01, -0.72) | -1.01 (-1.13, -0.89) | -0.82 (-0.96, -0.69) | -1.16 (-1.25, -1.06) | -1.15 (-1.30, -1.00) |
| Low SDI | -1.04 (-1.10, -0.98) | -1.08 (-1.16, -1.00) | -1.11 (-1.21, -1.02) | -1.04 (-1.15, -0.93) | -1.28 (-1.33, -1.23) | -1.26 (-1.33, -1.20) |
| Asia | -1.28 (-1.38, -1.19) | -1.78 (-1.88, -1.68) | -2.18 (-2.33, -2.04) | -1.61 (-1.70, -1.53) | -2.51 (-2.67, -2.35) | -2.59 (-2.70, -2.47) |
| China | -0.50 (-0.67, -0.32) | -1.61 (-1.73, -1.48) | -2.34 (-2.60, -2.07) | -1.25 (-1.38, -1.12) | -2.65 (-2.86, -2.43) | -2.75 (-2.92, -2.58) |
| Japan | -2.92 (-3.11, -2.72) | -2.95 (-3.16, -2.74) | -3.12 (-3.33, -2.90) | -2.93 (-3.14, -2.71) | -3.55 (-3.78, -3.32) | -3.43 (-3.61, -3.24) |
| South Korea | -1.65 (-1.91, -1.38) | -3.23 (-3.47, -2.98) | -4.47 (-4.65, -4.28) | -2.61 (-2.92, -2.31) | -4.95 (-5.17, -4.73) | -5.06 (-5.32, -4.79) |
| North Korea | -0.05 (-0.16, 0.05) | -0.48 (-0.52, -0.44) | -0.62 (-0.65, -0.60) | -0.33 (-0.37, -0.28) | -0.58 (-0.61, -0.56) | -0.74 (-0.77, -0.72) |
| Mongolia | -1.14 (-1.44, -0.84) | -1.26 (-1.60, -0.92) | -1.11 (-1.47, -0.75) | -1.25 (-1.57, -0.93) | -1.24 (-1.59, -0.89) | -1.48 (-1.80, -1.16) |
