# Supplementary material for: Burden and Future Trends of Gastric Cancer in 5 East Asian Countries From 1990 to 2036: Epidemiological Study Analysis Using the Global Burden of Diseases Study 2021
Source: JMIR Cancer. 2025 Sep 3;11:e74389. doi: 10.2196/74389 (PMC12408060; doi:10.2196/74389)
Supplement: Multimedia Appendix 5 [file cancer-v11-e74389-s005.docx]

Multimedia Appendix 5: Age-period-cohort analysis of prevalence, incidence, death rate.

Table of content

Figure S13. Age-period-cohort analysis of prevalence rate.

Figure S14. Age-period-cohort analysis of incidence rate.

Figure S15. Age-period-cohort analysis of death rate.

**Figure S13. Age-period-cohort analysis of prevalence rate.** **A: China, B: Japan, C: South Korea, D: North Korea, E: Mongolia.**


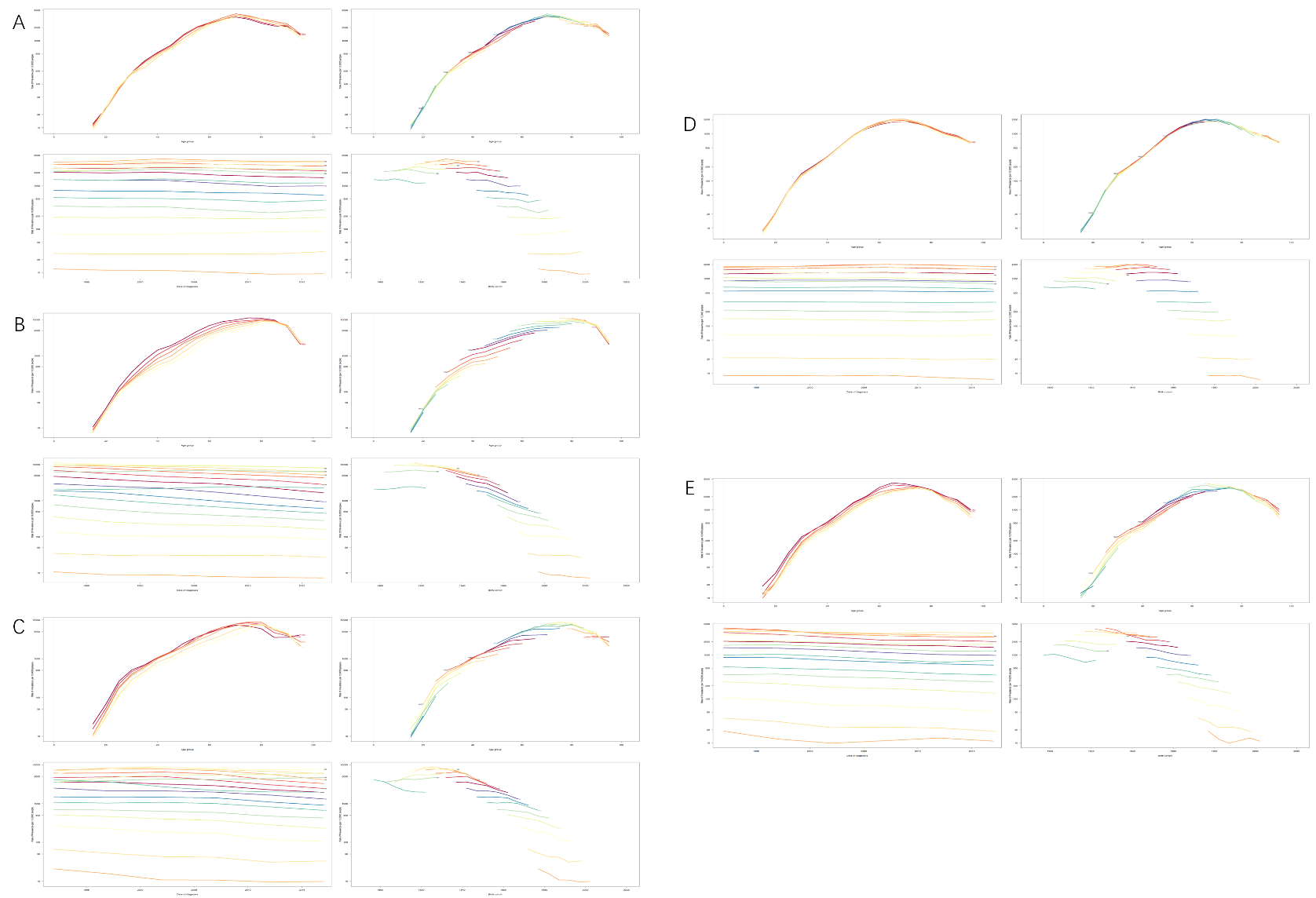


**Figure S14. Age-period-cohort analysis of incidence rate. A: China, B: Japan, C: South Korea, D: North Korea, E: Mongolia.**


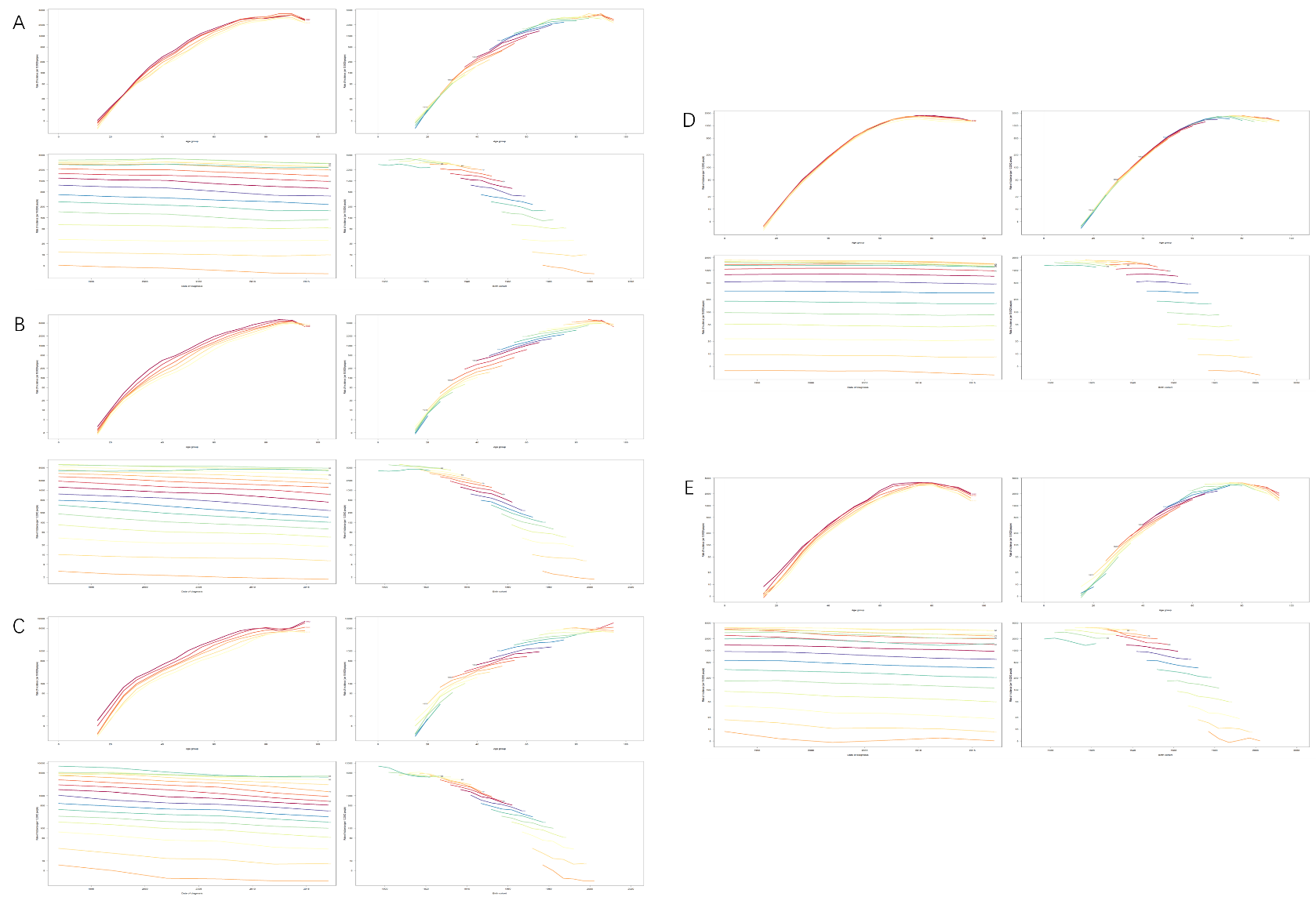


**Figure S15. Age-period-cohort analysis of death rate. A: China, B: Japan, C: South Korea, D: North Korea, E: Mongolia.**

**
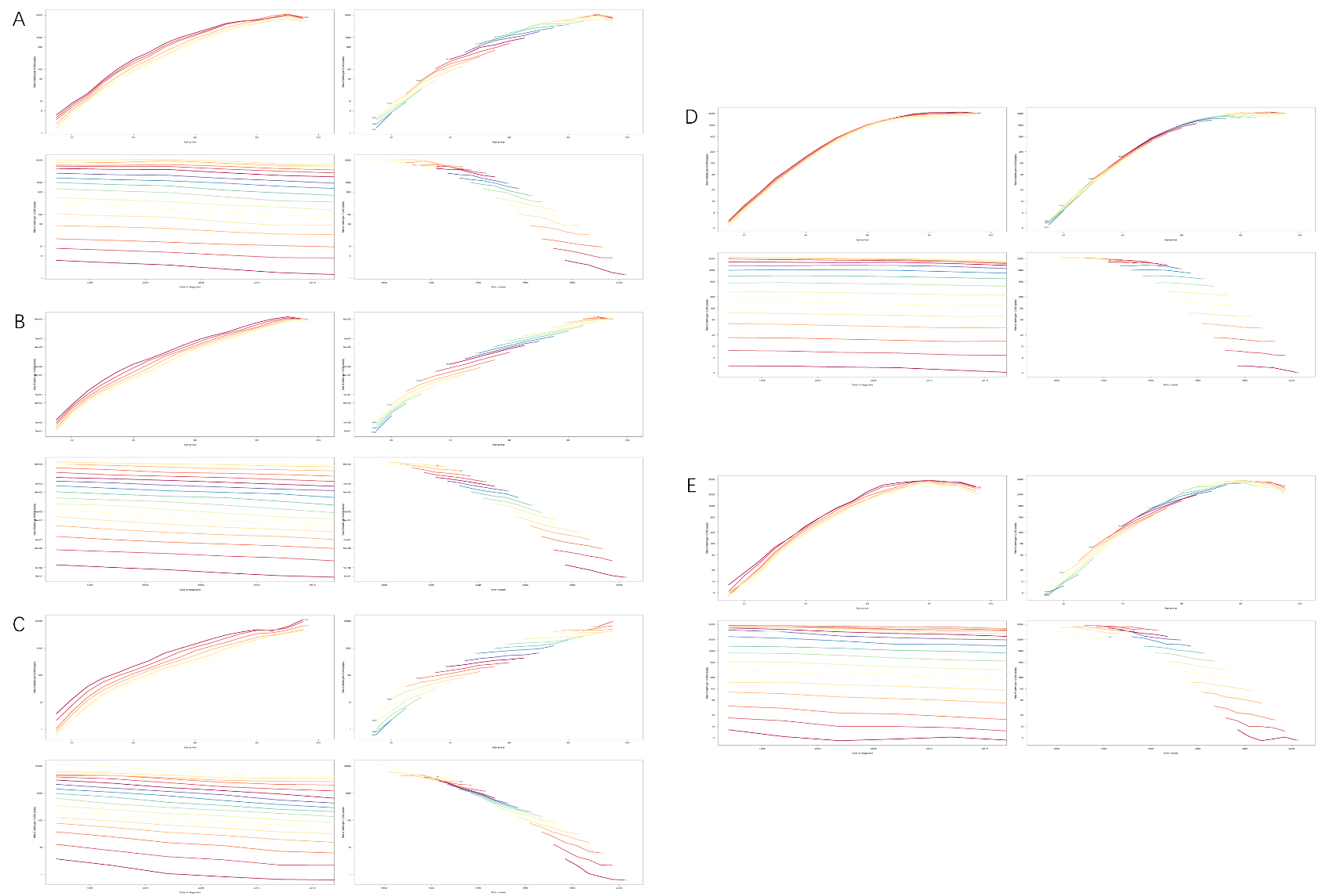
**
