# Supplementary material for: Burden and Future Trends of Gastric Cancer in 5 East Asian Countries From 1990 to 2036: Epidemiological Study Analysis Using the Global Burden of Diseases Study 2021
Source: JMIR Cancer. 2025 Sep 3;11:e74389. doi: 10.2196/74389 (PMC12408060; doi:10.2196/74389)
Supplement: Multimedia Appendix 6 [file cancer-v11-e74389-s006.docx]

Multimedia Appendix 6: ARIMA and BAPC prediction in the 5 East-Asian countries.

Table of content

Figure S16. ARIMA Prediction of ASPR.

Figure S17. ARIMA Prediction of ASMR.

Table S8. ARIMA prediction ASPR per 100000 people in the 5 East-Asian countries.

Table S9. BAPC prediction ASIR per 100000 people in the 5 East-Asian countries.

Table S10. ARIMA prediction ASMR per 100000 people in the 5 East-Asian countries.

**Figure S16. ARIMA Prediction of ASPR. A: China, B: Japan, C: South Korea, D: North Korea, E: Mongolia.**


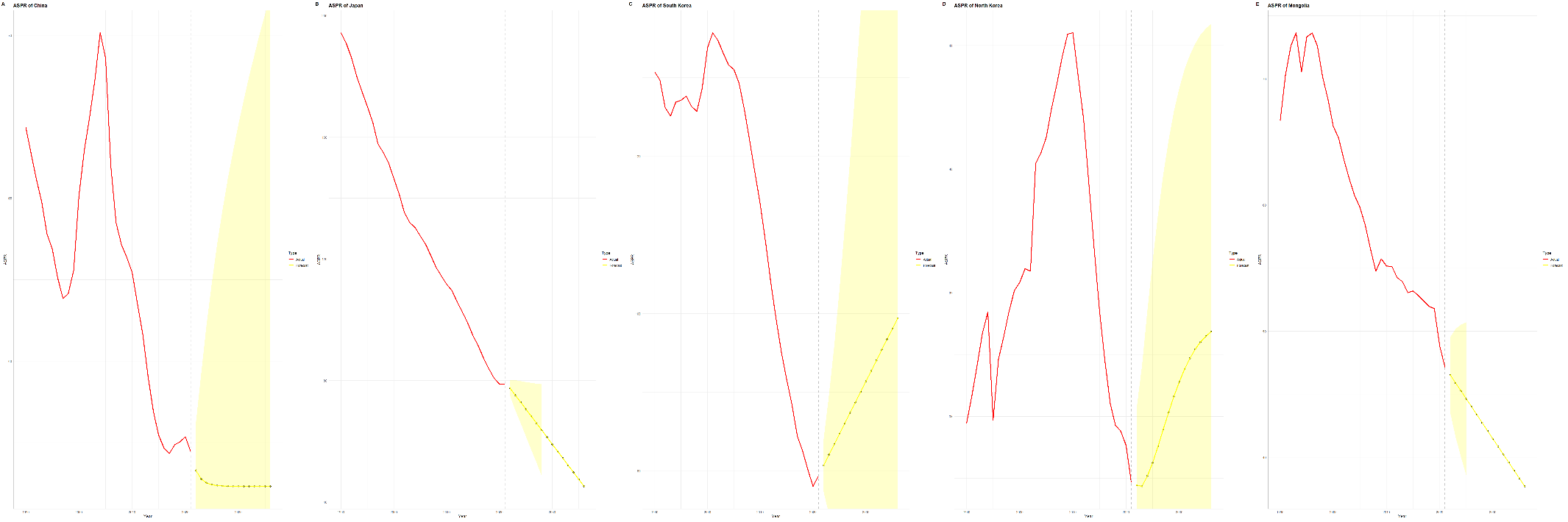


**Figure S17. ARIMA Prediction of ASMR. A: China, B: Japan, C: South Korea, D: North Korea, E: Mongolia.**


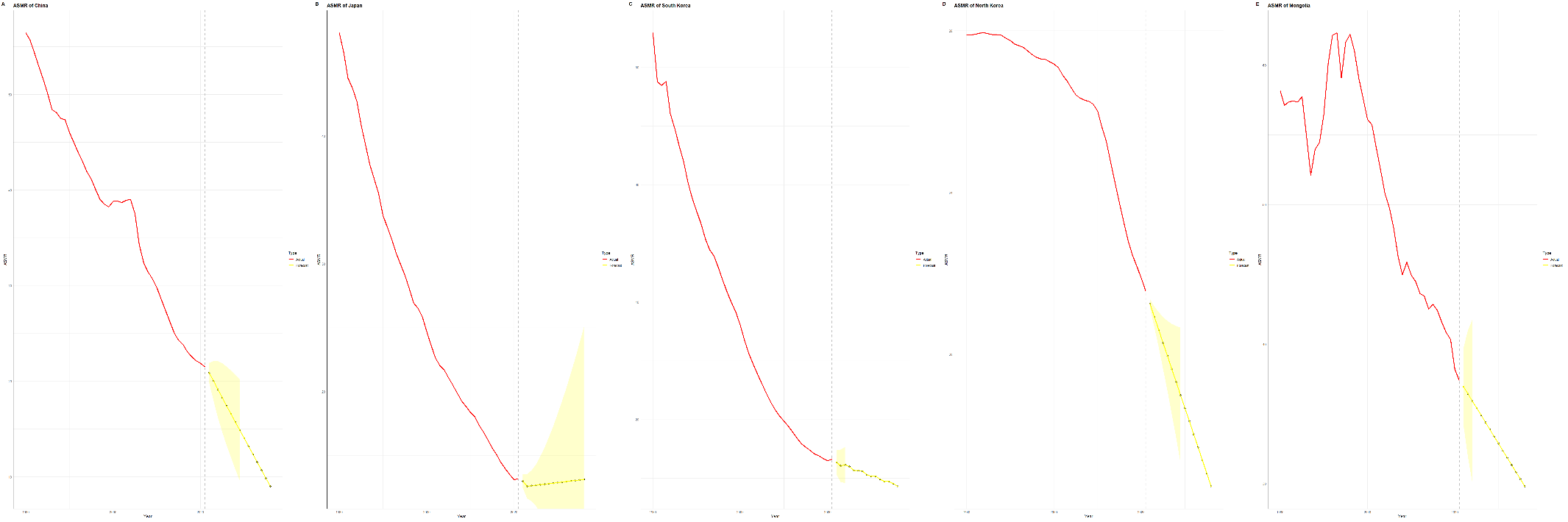


**Table S8. ARIMA prediction ASPR per 100000 people in the 5 East-Asian countries.**

| ASPR | China | Japan | South Korea | North Korea | Mongolia |
| --- | --- | --- | --- | --- | --- |
| 1990 | 67.17 | 194.37 | 130.74 | 37.94 | 66.68 |
| 2021 | 57.22 | 78.91 | 79.38 | 37.47 | 47.15 |
| 2030 | 56.15 | 59.04 | 91.40 | 38.28 | 41.49 |
| 2036 | 56.15 | 45.20 | 99.41 | 38.68 | 37.71 |

**Table S9. BAPC prediction ASIR per 100000 people in the 5 East-Asian countries.**

| ASIR | China males | China females | Japan males | Japan females | South Korea males | South Korea females | North Korea males | North Korea females | Mongolia males | Mongolia females |
| --- | --- | --- | --- | --- | --- | --- | --- | --- | --- | --- |
| 1990 | 67.95 | 30.38 | 95.38 | 41.19 | 110.13 | 46.12 | 42.72 | 18.88 | 74.85 | 43.38 |
| 2021 | 44.75 | 15.34 | 39.06 | 14.85 | 38.98 | 15.56 | 36.38 | 14.83 | 58.25 | 22.05 |
| 2030 | 41.54 | 13.58 | 34.22 | 11.76 | 33.67 | 13.32 | 33.18 | 12.73 | 51.32 | 16.41 |
| 2036 | 39.49 | 12.86 | 31.47 | 10.23 | 30.70 | 12.53 | 31.30 | 11.50 | 46.58 | 13.49 |

**Table S10. ARIMA prediction ASMR per 100000 people in the 5 East-Asian countries.**

| ASMR | China | Japan | South Korea | North Korea | Mongolia |
| --- | --- | --- | --- | --- | --- |
| 1990 | 46.05 | 33.76 | 55.44 | 27.76 | 56.43 |
| 2021 | 21.51 | 13.20 | 13.26 | 21.57 | 37.40 |
| 2030 | 14.05 | 12.89 | 10.35 | 18.67 | 32.87 |
| 2036 | 9.01 | 13.15 | 8.62 | 16.74 | 29.81 |
